# Trophic generalism in the winter moth: a model species for phenological mismatch

**DOI:** 10.1007/s00442-024-05629-5

**Published:** 2024-11-20

**Authors:** Jamie C. Weir

**Affiliations:** Ashworth Laboratories, Institute for Ecology and Evolution, University of Edinburgh, Charlotte Auerbach Road, Edinburgh, EH9 3FL United Kingdom

**Keywords:** Asynchrony, Resilience, Buffering, Polyphagy, Generalism

## Abstract

Climate change has the potential to disrupt phenological synchrony among interacting species that vary in their phenological sensitivity to temperature. The phenological synchrony observed between winter moth *Operophtera brumata* caterpillars and oak leafing in spring has become an emblematic test case of this phenomenon, with caterpillars seemingly advancing their phenology more than their host-plant. However, work on this trophic interaction—and on phenological mismatch more widely—routinely overlooks the potential for trophic generalism to buffer the negative effects of mismatch. In the largest study of its kind—using over 3500 individuals reared from egg to pupa—I tested the performance of winter moth caterpillars from four UK populations across nine host-plant species, and considered how adaptation to locally abundant host-plants may modulate performance in different populations. I found that caterpillars survive and grow well across a range of host-plant species, with some evidence of a host-plant by population interaction in performance. Contrary to widespread assumptions, oak seems a relatively poor host-plant species. Occupying a broad trophic niche may help consumers like the winter moth exploit a narrow phenological niche, whereby phenological variation among host-plant species buffers them against asynchrony with any one particular host-plant species. Determining the significance of trophic generalism in the ecology of consumers is a crucial first step towards assessing its role as a potential buffering mechanism and, hence, evaluating the true threat posed by mismatch.

**Supplementary Information:**

The online version contains supplementary material available at 10.1007/s00442-024-05629-5.

## Introduction

Over recent decades, rising global mean temperatures (IPCC 2021) have brought about directional shifts in phenology across a range of taxa (Cohen et al. 2018; Roslin et al. 2021; Thackeray et al. 2016). For many organisms, survival or successful reproduction can depend on timing a stage in their life history, such that it is synchronised with the timing of another species. For example, temperate fish species that fail to spawn at the correct time can find their offspring mismatched with the maximum abundance of a food supply, with negative consequences for recruitment to the population (Cushing 1969, 1990). Similarly, birds that mistime their breeding relative to the maximum availability of an ephemeral insect food supply can suffer reductions in offspring condition (Samplonius et al. 2016), individual fitness (Reed et al. 2013), and perhaps even population size (Both et al. 2006; Mclean et al. 2016; though cf. Samplonius et al. 2020). There is a growing concern among ecologists that divergent phenological responses to changing global temperatures in different species could lead to a temporal decoupling of many such timed interactions—‘phenological asynchrony’—with potentially catastrophic effects on populations and perhaps whole ecosystems (reviewed in Samplonius et al. 2020; Iler et al. 2021).

In a trophic context, phenological asynchrony is most likely to occur where a consumer exploits a single, ephemeral resource—this makes precise timing particularly important, and in theory, even small temporal misalignments can lead to reductions in fitness (Cushing 1967, 1969, 1990; Durant et al. 2007; Hjort 1914). Although most prior research focusses on simplified food chains (see Samplonius et al. 2020; Weir 2022), the complex structure of food webs in nature could reduce dependence on any one resource. Samplonius et al. (2020) found that in 74% of studies of phenological mismatch, both the consumer’s dependence on a resource and the ephemerality of that resource were assumed a priori and not directly tested. Very few studies explicitly test the extent to which consumers are generalists (Samplonius et al. 2020) and therefore the potential for generalism to buffer the negative impacts of asynchrony with any one trophic resource.

A plurality of studies which investigate the effects of phenological mismatch use the spring woodland tree/caterpillar/bird food-chain as a model system (Samplonius et al. 2020). Due to its abundance, the caterpillars of the winter moth *Operophtera brumata* (L.) are often taken as representative of the primary consumer level in this chain and it has become a standard study organism for phenological research (Charmantier et al. 2008; Cole et al. 2015, 2021; Hinks et al. 2015; Shutt et al. 2019; Visser et al. 2021). Winter moth caterpillars hatch as foliage appears on trees in early spring (Skinner 2009), ready to exploit this newly available food resource. Over the last few decades, evidence has accumulated suggesting that the fitness of winter moth caterpillars depends to a very large extent on precisely matching their phenology with that of their host-plants (Asch and Visser 2007; Buse et al. 1999; Van Dis et al. 2023; Dongen and Stefan 2006; Feeny 1970; Kerslake and Hartley 1997; Tikkanen et al. 1998; Tikkanen and Julkunen-Tiitto 2003; Tikkanen and Lyytikäinen-Saarenmaa 2002; Wint 1983). Caterpillars which hatch too early find themselves with no foliage to feed on and starve (Wint 1983); those hatching later are forced to feed on more mature foliage which has undergone structural changes and accumulated secondary chemicals reducing its nutritional value (Feeny 1968, 1970). The result is strong stabilising selection for close synchrony between the timing of caterpillar egg hatch and the timing of bud burst on their host-plants (Asch et al. 2007; Van Dis et al. 2023; Tikkanen and Julkunen-Tiitto 2003). However, framing the winter moth as reliant on synchrony with a single host-plant species (Table S1) may misrepresent its diet—in nature, we find a complex food web of many different interacting caterpillar and host-plant species.

Temperate spring-feeding caterpillars as a group are typically trophic generalists (Henwood et al. 2020; Maitland Emmet and Heath 1992; Porter 2010). Indeed, it has long been established that the winter moth is a highly polyphagous species (Allan 1979; Henwood et al. 2020; Maitland Emmet and Heath 1992; Meyrick 1895; Porter 2010; Stainton 1859; Stokoe 1948; Waring et al. 2017), with caterpillars recorded feeding on plants from 31 different genera across 15 families (Robinson et al. 2010). Occupying a broad niche can be optimal in uncertain environments (Levins 1968), and so, faced with uncertainty in various aspects of the environment (e.g., unpredictability of the developmental stage of any available leaves at the onset of spring), a generalist diet might have arisen in this species as a buffer against being mistimed with any one particular host-plant individual or species (Weir and Phillimore 2024).

However, trophic generalism in the winter moth is largely only mentioned in passing in the phenological literature (Table S1). Instead, the focus has mainly been on a single host-plant, English oak *Quercus robur* L. (Table S2; Roland and Myers 1987; Buse et al. 1998, 1999; Tikkanen and Lyytikäinen-Saarenmaa 2002; Tikkanen and Julkunen-Tiitto 2003; Dongen and Stefan 2006; Asch et al. 2007; Mannai et al. 2017; Kulfan et al. 2018). The reasons for this emphasis seem to be largely historical, tracing back to the earliest considerations of phenological synchrony in the winter moth (e.g., Thomson 1954; Feeny 1968; Varley et al. 1974). The focus on oak creates the impression of winter moth populations facing an ephemeral and moving resource peak in spring (young, nutritious oak foliage), as the oak itself responds plastically to temperature (Roberts et al. 2015). On the other hand, feeding on a wide range of host-plant species that vary in their leafing phenology may extend the period over which young leaves are available overall, serving as a buffer on trophic mismatch more broadly.

Despite the extensive literature on the winter moth, even the relative importance of very widespread and abundant alternative host-plant species, such as birch *Betula* spp., has rarely been considered (Table S2). Furthermore, even though there is considerable variation and turnover in flora throughout the Holarctic distribution of the winter moth, the previous studies considering local adaptation to host-plant are very limited in geographical scale (e.g., Kerslake and Hartley 1997; Tikkanen et al. 2000). Although genomic studies indicate large-scale dispersal and gene flow (Dongen et al. 1998; Legget et al. 2011), there are clearly populations in which performance is optimised on locally abundant host-plants (Belsing 2015; Kerslake and Hartley 1997; Tikkanen et al. 2000) suggesting strong selection in these areas. Since the flightless female winter moths exercise very little (if any) taxonomic discrimination with regard to the host-plant that their offspring will find themselves on, a broad diet could give this species flexibility, with local adaptation potentially fine-tuning and optimising performance at a local level. In addition, early instar caterpillars can disperse through “ballooning” if they find themselves in unsuitable conditions (Hunter 1990)—perhaps repeatedly in search of an optimal location (Edland 1971; Holliday 1977)—such that polyphagy could allow them to exploit neighbouring trees at a more suitable phenological stage, understory species, or even ground cover plants such as heather *Calluna* and blaeberry *Vaccinium*, if necessary (e.g., Belsing 2015; Kerslake et al. 1996)*.*

To test whether there is potential for trophic generalism to serve as a buffer against phenological mismatch with host bud burst, I quantified the performance of winter moth caterpillars across a range of common and widespread host-plant species. Understanding the performance effects of different diets is a vital first step in assessing the capacity for alternative host-plants to act as buffers against mismatch. (**Aim A**) I conducted an extensive assay of performance across several metrics on nine abundant and potentially significant host-plant species, using 3600 caterpillars—the largest such experiment to date. (**Aim B**) Additionally, to test for geographical divergence in performance across host-plant species (consistent with local adaptation), I assayed livestock sampled from four populations across Great Britain. I found that although performance varied substantially, a wide range of host-plants could be utilised effectively. In contrast to expectations, oak proved a relatively poor host-plant, in terms of caterpillar performance. I consider the implications of these results for the resilience of temperate woodland food webs under climate change (of which the winter moth forms a crucial part), and for the impacts of phenological asynchrony on trophic generalist versus specialist taxa more broadly.

## Materials and methods

### Source populations

I obtained winter moth ova from four populations across Great Britain (Table 1). I collected females from the Edinburgh population using trunk traps, modelled on those described by Varley et al. (1974). I collected a total of 165 females between 25 Nov 2019 and 8 Jan 2020, across 72 traps. Entomologists located near the three other sites each provided me with a minimum of fifteen female winter moths from each (see Table 1). Individuals from these populations were collected manually by searching trunks after dark by torchlight. These populations were selected due to their geographical spread and variation in local habitat types.

**Table 1:** UK collection sites of winter moth livestock used in the host-plant assay, with a description of the local flora

	Site	Co-ordinates	Habitat characteristics and Alt
	**Buckinghamshire** Hill Farm Cottage, Buckingham (VC 24)	51.978946°N −0.983623°E	Hamlet surrounded by grazing pasture. Small garden orchard of apple, pear, plum, cherry, fig and apricot. Dry area, with extensive hedges of hawthorn, maple, ash, blackthorn 110 m
	**Devon** Dart Valley Nature Reserve, Poundsgate (VC 3)	50.530946°N −3.849855°E	Ancient, damp, primarily oak woodland, situated in heathland 280 m
	**Edinburgh** Hermitage of Braid LNR, Edinburgh (VC 83)	55.919501°N −3.197014°E	Exposed patch of mature oaks on the edge of a large mixed woodland, adjacent to grassland. Sycamore abundant throughout 105 m
	**Suffolk** Ipswich Golf Course, Ipswich (VC 25)	52.042964°N 1.215717°E	Sheltered site at the edge of a mixed woodland of oak, birch, sycamore, Scots pine. Surrounded by dry heathland and short-cropped grassland 20 m

### Rearing methodology

After collection, females were placed individually in 75 × 25 x 25 mm glass tubes with a wad of cotton at the bottom to act as an egg-laying medium. Females from all sites were stored at approx. 5 °C in complete darkness and allowed to lay freely. Approximately 1 month later, all tubes were examined and the dead females were removed. Ova from a total of 126 females from the Edinburgh site, 15 from Buckinghamshire, 14 from Devon, and 19 from Suffolk were obtained.

When foliage became available in spring, ova were removed from cold storage and placed at room temperature (approx. 20 °C) to stimulate egg-hatching. A subset of ova, sampled from across all broods, were removed concurrently and allowed to hatch. Exposure to relatively high temperatures helped ensure individuals hatched at the same time, despite inter- and intra-brood variation in the temperature requirements for eclosion. Caterpillars were assigned at random from each brood to each treatment group, and subsequently to each rearing culture within that treatment group.

**Fig. 1:**
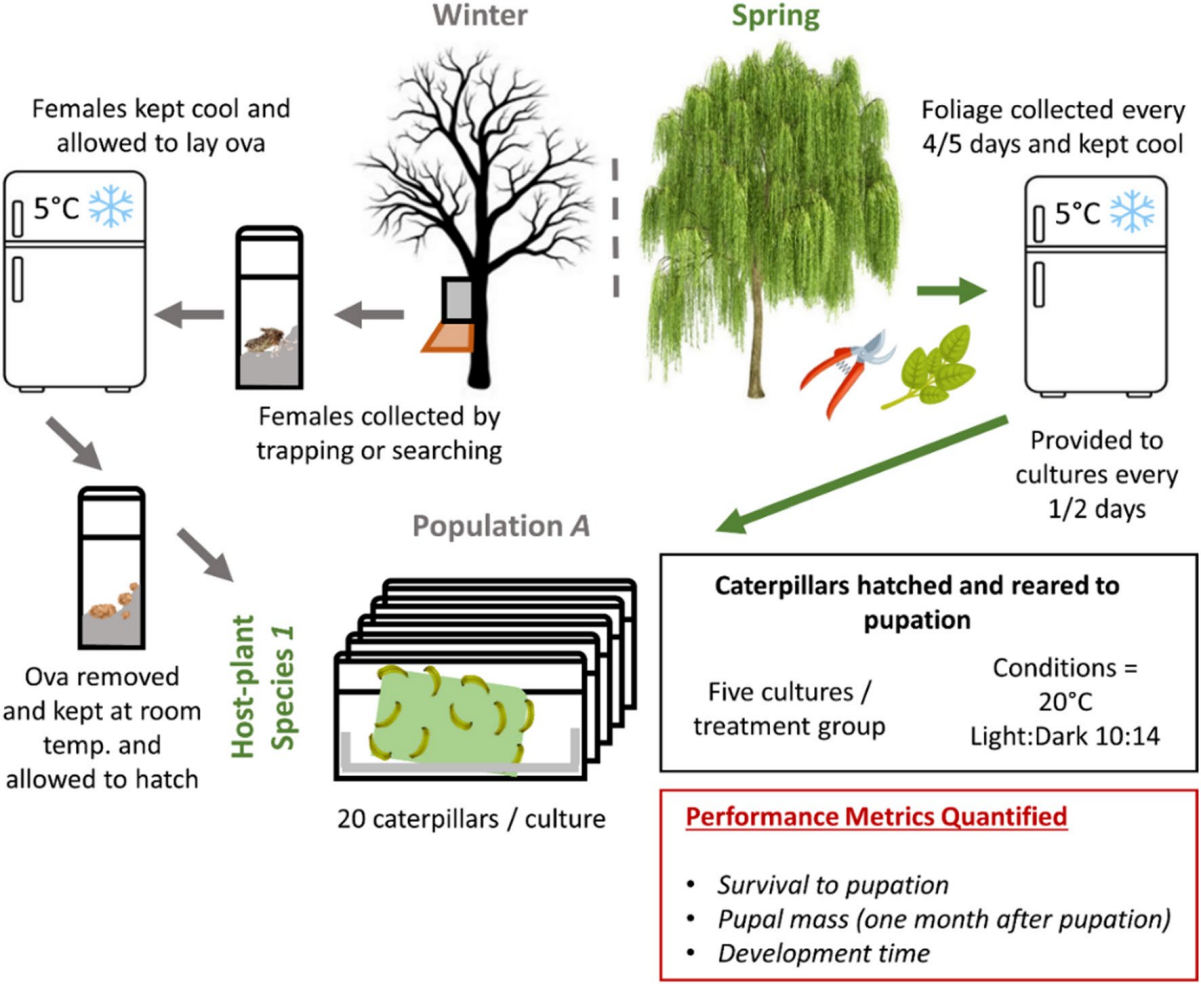
Schematic of the experimental design. Winter moth livestock from four British populations were reared to pupation on foliage from nine different host-plant species. Caterpillars were reared in cultures of 20 individuals, assigned to each culture randomly across broods. Performance in each host-by-population treatment was quantified on three metrics: survival, pupal mass attained, and development time (time from egg hatch to pupation)

Caterpillars were reared in mixed-brood groups of 20 individuals (a “culture”), first in small 75 × 50 x 15 mm transparent plastic containers and then, at around the third instar, in larger 500 ml disposable plastic containers (Fig. 1 and S1; for a discussion of the mixed-brood culture rearing method, see Appendix 2). Cultures were established concurrently from caterpillars hatched in the previous 24 h. The rearing containers were lined with white absorbent paper towels. Freshly excised food was placed in each container and examined daily to check its condition and how much remained. Typically, it was replaced daily, no less than every second day (Fig. 1). Caterpillars were provided with an excess of plant material at all times, such that the quantity of food was never a limiting factor to growth. The tissue lining of the container was replaced each time new food was provided. Caterpillar rearing cultures were maintained together at room temperature (approx. 20 °C) with a 10:14 light:dark regime.

At the completion of their development, the caterpillars pupated in the tissue at the base of the container. After all larvae had pupated, excess host-plant material was removed and the containers were stored at room temperature. One month after pupation, pupae were removed, laid out on cotton for emergence in a sealed plastic container, and stored outdoors under a canopy at ambient environmental temperature (Stirlingshire; 56.069°N, -3.767°E).

### Host-plant assays

The aims of this experiment were to determine:

(**Aim A**) how caterpillar performance differed among nine common host-plant species;

and,

(**Aim B**) if performance differed across the different geographical populations in a manner consistent with local adaptation.

Nine known host-plant species of the winter moth (Robinson et al. 2010) which are common and widespread in Britain were selected for use in the assays: alder *Alnus glutinosa*, apple *Malus domestica*, birch *Betula pendula*, cherry *Prunus avium*, hawthorn *Crataegus monogyna*, oak *Quercus robur*, sallow *Salix caprea*, sycamore *Acer pseudoplatanus*, and willow *Salix alba* (Fig. S2). Criteria for selecting these species were a combination of logistical considerations (e.g., the availability of sufficient trees for foliage collection) and hypothesised ecological or evolutionary importance. In the context of their potential to act as a buffer on mismatch, host species which are abundant and widespread are most likely to be significant. Furthermore, I wanted to compare performance on oak (the standard study species) with very common understory species which might easily be accessed by asynchronously hatching larvae (e.g., alder, birch, and hawthorn) and those known to host a wide range of insect herbivores (e.g., sallow and willow). In considering local differentiation in performance, it was also important to include species which have been found to be significant hosts in European populations but which have not been studied extensively in Britain (e.g., sycamore, see Tikkanen et al. 1998; or cherry, see Tikkanen et al. 2000).

One hundred larvae from each source population were randomly assigned from across broods to each of the nine host-plant species, at 20 larvae per rearing culture (Fig. 1), totalling 3600 caterpillars. Since caterpillar fitness can vary depending on foliage age (Asch et al. 2007; Tikkanen and Julkunen-Tiitto 2003), the calendar start date of each host-plant treatment was staggered, such that for each host-plant species, the experiment began when a sufficient number of individual trees could be sampled in the field at an appropriate phenological stage (Table S3). This also ensured that host phenology and species were not confounded, for example by collecting from unusual late-leafing hawthorn trees (an earlier species, on average) to coincide with leaf collection on a typically later-leafing species, such as oak.

I started collecting leaves from each species when buds were fully open and the shape of the developing leaves were recognisable. In oak, for example, these young leaves were several centimetres long, bright green, and curled (Fig. S4). Fresh foliage was collected from 12 individual trees of each species every 4–5 days as cut sprigs 15 cm long and stored in airtight plastic bags at approx. 5 °C in a tabletop refrigerator (Russell Hobbs RHCLRF17) until required for feeding. Foliage was collected from trees near Falkirk (Stirlingshire; 56.069°N, −3.767°E) and Kincardine (Fife; 56.057°N, −3.613°E). It should be noted here that, although this foliage appears ‘young’, feeding can commence in a natural setting as soon as soon as the scales of the developing host buds begin to open, and newly hatched caterpillars can burrow inside. The age of the foliage being provided to the caterpillars in this experiment should therefore be borne in mind when interpreting the results (see Discussion).

To minimise the effects of individual variation in leaf properties *within a host species* (see for example Laitinen et al. 2005; Lindroth 2012; Kos et al. 2015), leaves from across all the sampled tree individuals were randomly assigned to each rearing culture, such that larvae always had access to foliage from a range of different host-plant individuals belonging to the same species.

The performance of caterpillars in each treatment group was quantified by measuring:

- The survival of each individual from hatch to pupation;
- The final pupal mass attained by each individual 1 month after pupation (measured using a Mettler AJ50 balance, to 0.0001 g);
- The time taken for caterpillar development from egg hatch to pupation (Fig. 1).

Since larvae were reared in groups, it was not possible to relate each of these values to a specific individual.

### Statistical analyses

Analyses of larval performance were conducted in *R* v.4.0.3 using MCMCglmm (Hadfield 2010). Survival to pupation (“Surv”, binomial response with binary outcome), pupal mass (“Mass”, Gaussian), and development time (“Dev Time”, Gaussian) were each modelled separately (Table 2). Models included the following random terms:

*Host-plant species*, to allow estimation of the overall differences in performance among host-plants across all populations (Aim A).

*Population*, to allow estimation of differences in the average performance of different populations across all host-plants. If performance varied significantly by population, this may suggest systematic problems with the experimental design, e.g., livestock from one area experiencing different conditions.

*Host-plant species by Population interaction*, to allow estimation of population-specific differences in performance on different host-plants (Aim B), and examination of whether geographical divergence in performance between populations is consistent with local adaptation.

In each model, Rearing Culture was fitted to control for differences between each culture. In the Mass model, Sex was included as an additional random effect, because mass varies by sex but could only be determined by sexing the pupae, not larvae, and it could therefore not be included in other models.

**Table 2:** Modelling the effects of host-plant on winter moth caterpillar performance, measured as survival to pupation, pupal mass, and development time

Response variable	Random effect term	Justification
Surv/dev. time	Host-plant	Tests whether performance is consistently higher on certain host-plant species across all four populations
	Population	Tests whether performance is consistently higher in caterpillars from certain populations across all host-plant species. This shows whether, for example, individuals from some populations are performing consistently better in the common garden environment (e.g. perhaps populations closer to that site would perform better than those collected from farther away, due to, e.g., adaptation to weather conditions, clines in host-plant traits, etc.)
	Host-plant:population	Tests whether performance on certain host-plants is population specific, i.e. do caterpillars from one population perform better on a particular host-plant species that those from another population
	Rearing culture	Tests whether performance is consistently higher in individuals reared in the same captive environment, a “culture”
Mass	Host-plant	As above
	Population	As above
	Host-plant:population	As above
	Sex	Tests whether performance is consistently higher in one sex compared with the other
	Rearing culture	As above

Justification gives the hypothesis tested by each term. A significant Host-plant by Population interaction would be consistent with local adaptation. A significant effect of population, on the other hand, would perhaps indicate flaws in the experimental procedure, particularly if the performance was highest in the population most proximate to the rearing site (Edinburgh). Colon (“:”) indicates an interaction term in *R* statistical modelling syntax

Because individual caterpillars were reared in mixed-brood cultures, it was not possible for most models to include a random effect of brood. To assess the potential of this unaccounted-for source of pseudoreplication to bias inferences, I conducted extensive simulations, manipulating levels of within- and among-brood variance and assessing the impact on model estimates (bias and precision) and on false-positive rates and power. The simulations suggest that the experimental design generates conservative estimates for the variance and significance of the focal model parameters, and is therefore robust in terms of addressing the stated aims of this study (see Appendix 2).

All models were run for 1,500,000 iterations with a 500,000 burn-in and thinning every 100 iterations. In the binomial model for survival, default priors were used for the fixed effects (mean = 0, with a large variance), inverse-Wishart priors for the random effects, and the residual variance was fixed. In the remaining Gaussian models, the default priors were used throughout.

Variance components were estimated on the link scale for each model. Using the posterior distributions of survival, pupal mass, and development time, I also estimated the rate of development (mg/day) and projected absolute individual fitness (eggs/female) in each treatment group (for detailed explanation, methodology, and derivation, see Appendix 1).

### Detecting local adaptation

Two different criteria have been advanced for detecting local adaptation: (1) that a local genotype performs better than any other genotypes in that local environment (“local vs foreign”); and (2) that a local genotype performs better in its local environment than in foreign environments (“home vs away”). Where a genotype is locally adapted, both of these criteria will often be fulfilled. However, Criterion 2 is likely to be misleading where there are significant underlying differences in average fitness in different environments, and it should not be regarded as a definitive test (Kawecki and Ebert 2004). In the common garden set-up of this experiment the ‘environments’ are alternative host-plants. Comparison of the results of the model outputs and the characterisations of the local flora of each population (Table 1) allow for an evaluation of these criteria in this study. For example, we would expect a locally adapted genotype derived from a predominantly oak woodland site to show relatively higher fitness on oak than genotypes derived from other populations where oak is less abundant, under Criterion 1.

## Results

### (Aim A) Caterpillar performance and fitness across host-plant species

#### Survival

**Fig. 2:**
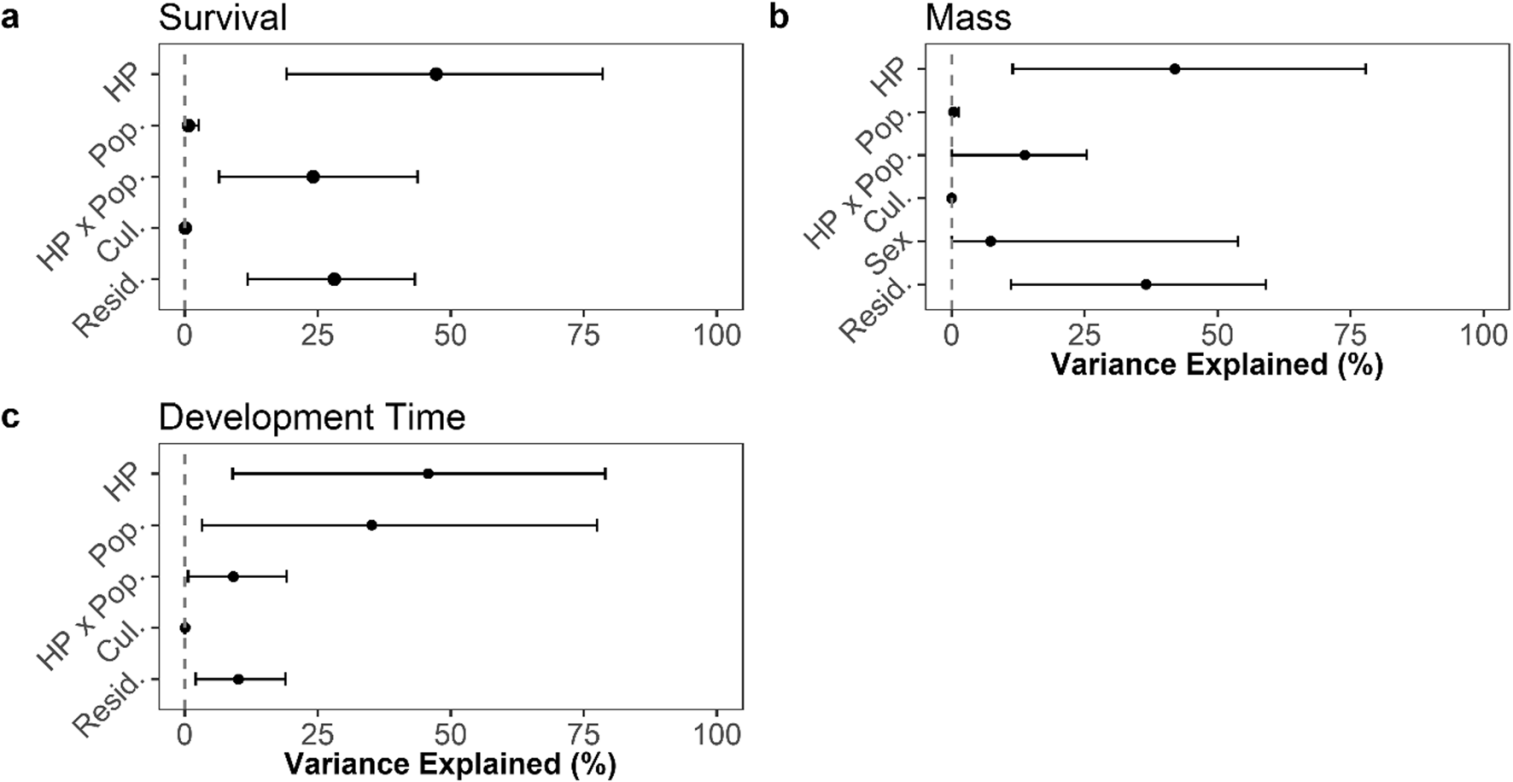
Variance components of caterpillar performance across host-plants and populations. Relative percentage (+/− 95% CIs) contribution of different effects to overall variance explained by **a** survival, **b** development time, and **c** mass models (Model 4). *Cul* rearing culture, *Resid.* residual variation, *HP* host-plant species, *Pop.* population, *HP x Pop.* host-by-population interaction effect, *Sex* individual sex. Estimates shown on the link scale

**Fig. 3:**
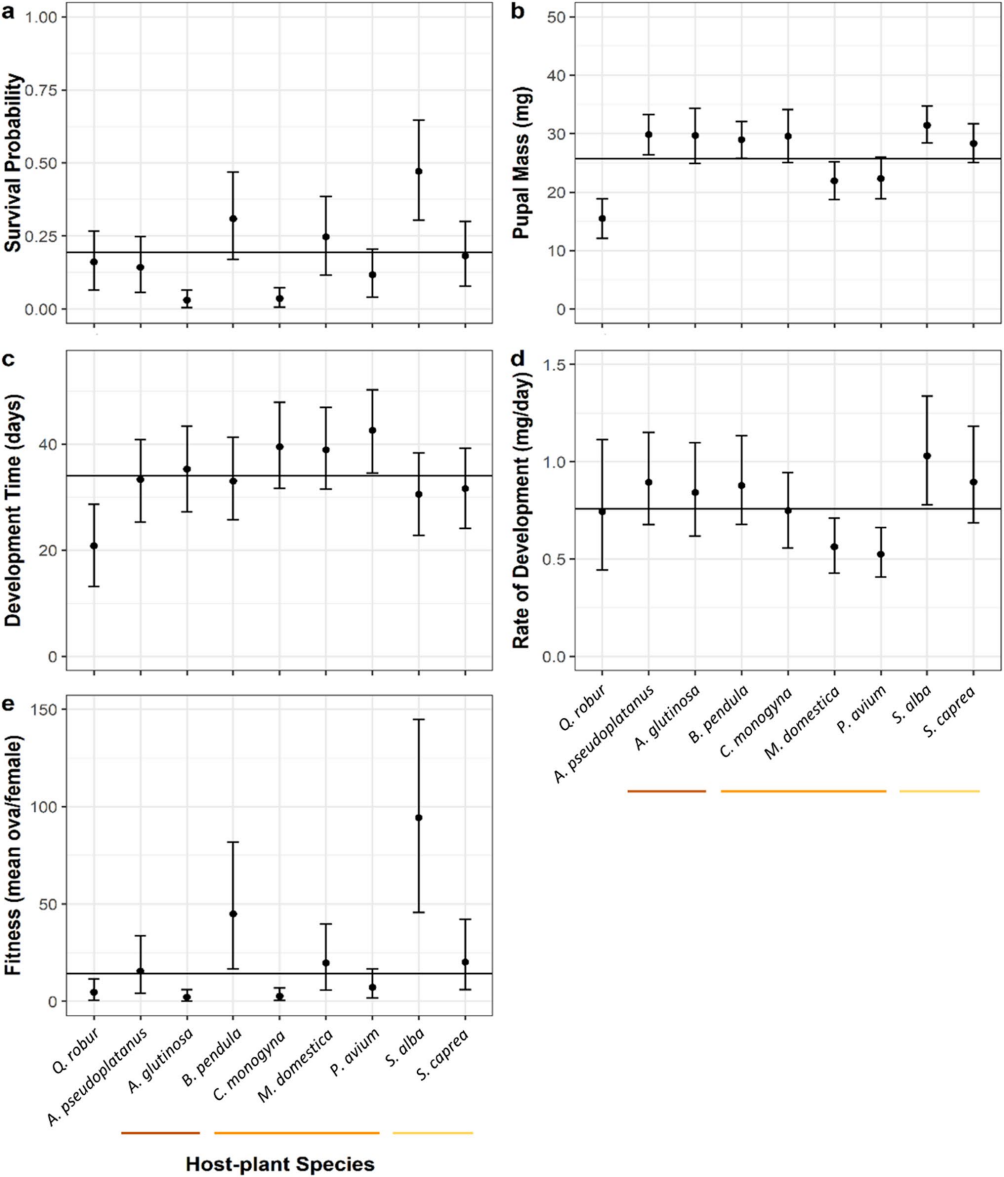
Overall winter moth performance on different host-plants, across all sampled populations. Performance quantified as **a** survival probability, **b** pupal mass, **c** development time, **d** rate of development, and **e** estimated fitness. Mean estimates and 95% credible intervals shown. Global mean for each performance metric shown by solid line. Host-plants in the same taxonomic family are grouped by a coloured underline: oak *Quercus robur* (FAGACEAE); sycamore *Acer pseudoplatanus* (SAPINDACEAE); alder *Alnus glutinosa* and birch *Betula pendula* (BETULACEAE); hawthorn *Crataegus monogyna*, apple *Malus domestica*, and cherry *Prunus avium* (ROSACEAE); sallow *Salix caprea* and willow *Salix alba* (SALICACEAE)

The mean individual probability of survival to pupation across all treatment groups and populations was 0.18. Survival probability varied significantly among host-plant species (Table 3; Fig. 2; 47% of variance on the link scale, 95% Credible Intervals: 19, 79). Relative to oak (0.16, CIs: 0.06, 0.27), survival was lower on alder (0.03, CIs: 0.00, 0.06) and hawthorn (0.04, CIs: 0.00, 0.07) and markedly higher on willow (0.47, CIs: 0.30, 0.65), but did not differ significantly between oak and the remaining host species (Fig. 3).

**Table 3:** Summaries for survival, pupa mass, and development time models. Colon (“:”) indicates an interaction term in *R* statistical modelling syntax. Means are given with 95% credible intervals

	Coefficient/variance (mean and CIs)	Effective sample size
**SURVIVAL MODEL**		
**Fixed terms**		
Intercept	−2.29 (−3.35, −1.30)	10,000
**Random terms**		
Rearing culture	0.0005 (0, 0.0025)	1367
Population	0.046 (0, 0.091)	10,000
Host-plant	2.09 (0.27, 5.15)	9457
Population:host-plant	0.87 (0.33, 1.50)	4341
**MASS MODEL**		
**Fixed terms**		
Intercept	26.3 (19.6, 31.5)	10,000
**Random terms**		
Rearing culture	0.009 (0, 0.035)	9041
Population	0.68 (0, 0.96)	10,000
Host-plant	33.4 (5.0, 79.5)	10,000
Population:host-plant	9.3 (3.7, 16.4)	2467
Sex	1695.0 (0.0, 77.4)	10,000
**DEV. TIME MODEL**		
**Fixed terms**		
Intercept	33.9 (24.6, 42.3)	10,000
**Random terms**		
Rearing culture	0.008 (0, 0.027)	5620
Population	68.7 (1.4, 186.9)	1210
Host-plant	50.8 (10.2, 116.9)	10,000
Population:host-plant	8.6 (3.2, 15.1)	10,000

#### Pupal mass

Mean pupal mass across all treatment groups 1 month after pupation was 26.2 mg (sd = 7.6). Female pupae (26.8 mg, sd = 8.1) were slightly heavier on average than those of males (25.6 mg, sd = 7.0). Pupal mass varied substantially among host-plant species (Table 3; Fig. 2; 42% of variance, CIs: 12, 78). Pupal mass was significantly higher on all other host-plant species than on oak (Fig. 3). Host-plant species fall into three discrete groups with regard to pupal mass attained, with apple and cherry being intermediate between oak and all the remaining species (Fig. 3).

#### Development time

Mean development time across all treatment groups was 32.1 days (sd = 6.5). Development time varied substantially across host-plant species (Table 3; Fig. 2; 46% of variance, CIs: 9, 79). Development time on oak did not differ significantly from the mean, but it was significantly shorter than on some other host-plants, such as hawthorn, apple, and cherry (Fig. 3).

#### Estimated rate of development

Mean rate of development across all treatment groups was 0.76 mg/day (CIs: 0.50, 1.09). On a majority of host-plant species, the estimated rate of development does not depart significantly from the mean (Fig. 3). However, rates were significantly higher than average on willow 1.03 mg/day (CIs: 0.78, 1.34) and lower on apple (0.56, CIs: 0.43, 0.72) and cherry (0.52, CIs: 0.41, 0.66). Notably, development rate on oak (0.74, CIs: 0.44, 1.11) did not differ significantly from any of the other host-plant species (Fig. 3).

#### Fitness

The arithmetic mean of estimated fitness (projected eggs per female per treatment, see Appendix 1) across all treatment groups was 22.2 (sd = 31.8). Fitness was significantly higher than average on birch (44.8, CIs: 16.7, 81.9) and willow (94.3, CIs: 45.6, 144.9), and lower on oak (4.7, CIs: 0.5, 11.6), alder (2.2, CIs: 0.3, 5.9), and hawthorn (2.7, CIs: 0.4, 6.8). Relative to oak, fitness was higher on birch and willow—two abundant and widespread species (Fig. 3).

### (Aim B) Geographical divergence in caterpillar performance

**Fig. 4:**
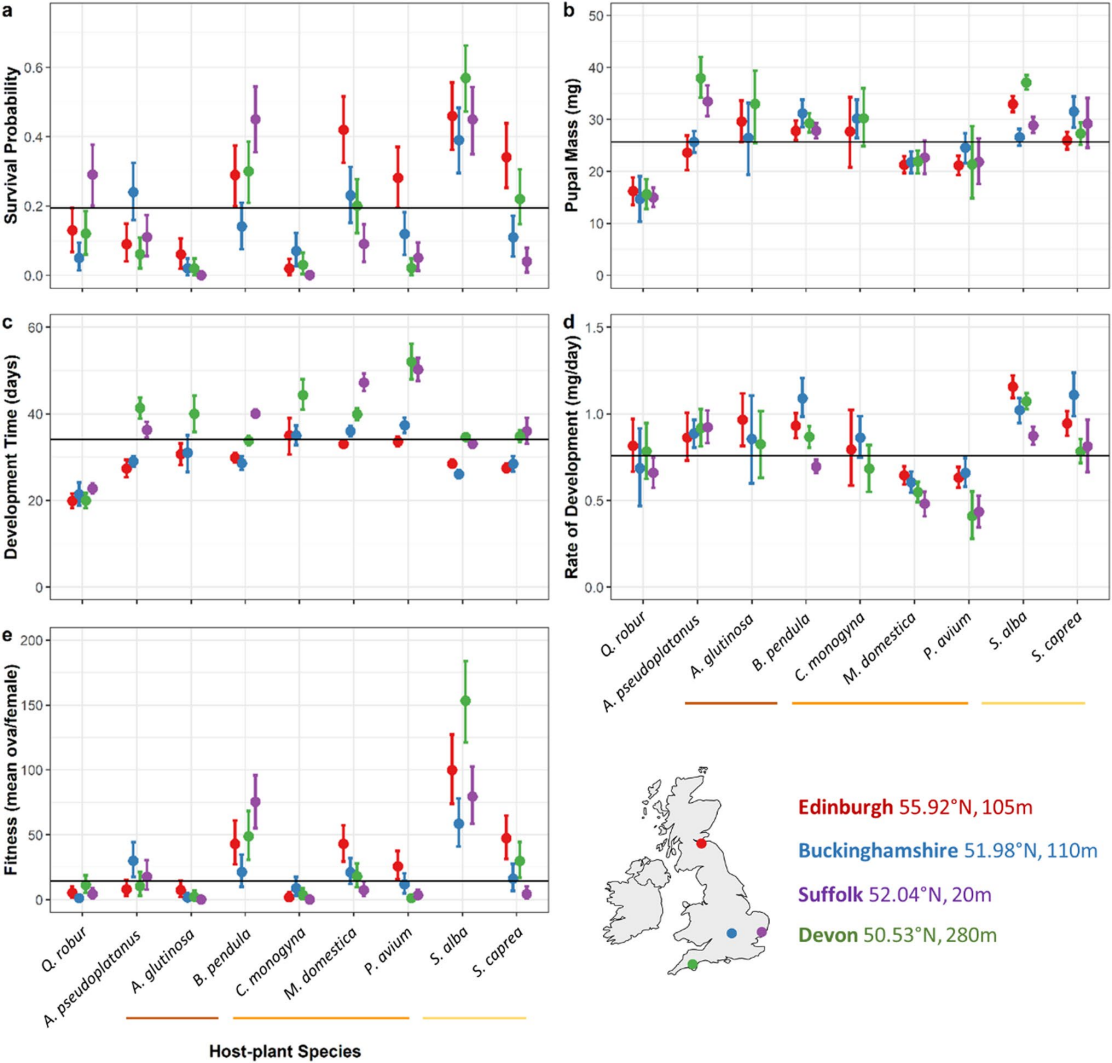
Winter moth performance in each host-plant by population treatment group. Performance quantified as **a** survival probability, **b** pupal mass, **c** development time, **d** rate of development, and **e** estimated fitness. Mean estimates and 95% credible intervals shown. Global mean for each performance metric shown by solid line. Host-plants in the same taxonomic family are grouped by a coloured underline: oak *Quercus robur* (FAGACEAE); sycamore *Acer pseudoplatanus* (SAPINDACEAE); alder *Alnus glutinosa* and birch *Betula pendula* (BETULACEAE); hawthorn *Crataegus monogyna*, apple *Malus domestica*, and cherry *Prunus avium* (ROSACEAE); sallow *Salix caprea* and willow *Salix alba* (SALICACEAE). Inset map shows colour-coded populations, with latitude and altitude

Source population main effects explain a sizeable portion of variation in development time (Table 3; Fig. 2; 35%, CIs: 3.2, 77.5), which is generally more prolonged in livestock sourced from the Devon and Suffolk populations (Fig. 4 and S3). Although development time is generally more protracted in these two populations, between-population differences in rate of development are less obvious and less pronounced (Fig. 4). For the remaining response variables, the source population variance posterior means and upper credible intervals were quite small.

The host-by-population interaction term explained a substantial quantity of variation in survival (24%, CIs: 6, 44), pupal mass (14%, CIs: 0, 25), and development time (9%, CIs: 0, 19) (Table 3; Fig. 2). This indicates differences in performance on the same host-plant species among source populations which may be a result of genetic divergence and local adaptation. However, although performance on a given host-plant varied considerably among populations, there was no obvious indication of local adaptation when comparing performance with the flora of each site (Table 1).

## Discussion

In this large-scale experimental study of performance, assaying 3600 caterpillars across nine host-plant species, I found that winter moth caterpillars are highly polyphagous trophic generalists. Although performance varied across host-plants, caterpillars survived and grew well on a wide range of species. There were clear differences between populations in terms of the performance on different host-plants, although this did not obviously correlate closely with the local flora of each site. Oak proved to be an unexceptional host-plant, running counter to the common framing of English oak (*Q. robur*) as the most significant host-plant species for the caterpillars of this moth in the wild (Table S1) and its use as the model host-plant in studies of phenological asynchrony (Table S2). Indeed, variation in performance among hosts, as shown here, may imply substantial variation in the fitness effects of asynchrony across host-plant species. These results present a crucial first step in evaluating the role of trophic generalism as a potential buffer against phenological mismatch. In the case of the winter moth, the evidence presented here is consistent with the hypothesis that a broad diet might confer resilience in the face of asynchrony induced by climate change. The clear next step to verify this hypothesis is to conduct direct tests in a natural setting.

### The significance of oak as a host-plant of the winter moth

If oak is at all notable as a host-plant in the results presented here, it is as a relatively poor one. First, I found that developmental time—a well-established, though inconsistent, signifier of environmental stress in Lepidoptera (Awmack and Leather 2002; Goulson and Cory 1995; York and Oberhauser 2002)—is considerably shorter on oak than on all other host-plant species, and no compensatory effect of rate of development was observed (Figs. 3 and 4). Second, pupal mass is significantly lower on oak (Fig. 3), consequently producing females with a greatly reduced fecundity (Appendix 1). Mean estimated fitness lags far behind many species, at the third lowest level attained in this experiment (Fig. 3). Indeed, fitness is markedly higher on some of the other common, widespread host-plant species, such as birch (four times higher) and willow (eight times higher).

The limited experimental work conducted prior to this study reports mixed results with respect to winter moth caterpillar performance across host-plants (Table S2). In some cases, performance is indeed highest on oak (O’Donnell et al. 2019; Vanbergen et al. 2003), and even on evolutionarily novel oak *Quercus* species (e.g., N. American *Q. rubra*; Embree 1965, 1970)*.* In other instances, alternative host-plant species prove equally suitable or better—however, in a plurality of multi-species studies, oak results in average or mixed caterpillar performance, across a range of metrics (Cuming 1961; Kirsten and Topp 1991; Tikkanen et al. 2000; Tikkanen and Lyytikäinen-Saarenmaa 2002; Wint 1983). However, performance has been found to vary substantially even in comparisons between oak species (Ján Kulfan et al. 2018; Mannai et al. 2017; Wesołowski and Rowiński 2008). In the classic study by Wint (1983), which is often cited as evidence for the importance of oak, the picture was also mixed (pupal mass was highest on oak, but survival was higher on almost all other host-plant species assayed). However, in these studies, performance across hosts is often inferred or quantified incidentally to the main aim of the study, employing considerably smaller sample sizes, fewer host species, and limited geographical comparisons. Here, I have presented firm evidence of the extent—and potential ecological consequence—of trophic generalism in this species.

Wint (1983) found considerable variability in performance both among years and across the growing season, to the extent that it was difficult to draw clear conclusions about the most suitable host species. It is certainly possible, therefore, that the particularly poor performance of oak in this experiment is a result of year-specific growing conditions, and would not be repeated to the same extent in other years. Indeed, the low captive-reared performance on oak is particularly striking when we consider that this does not include mortality due to parasitism, predation, and other extrinsic factors (Holliday 1977; Varley et al. 1974). The experimental design used in this study may have contributed to this low performance. For example, when reared in close captivity winter moth caterpillars can exhibit cannibalism, increasing overall levels of mortality in each treatment. Although this problem would apply across all host-plant treatment groups, it may exaggerate low performance on poor hosts where under-nourished caterpillars resort more readily to cannibalism. In addition, much higher survival has been found in studies where caterpillars have been reared in cages on living host-plant tissue (O’Donnell et al. 2019; Vanbergen et al. 2003), avoiding potential changes in leaf secondary chemistry occurring after foliage excision. However, outdoor rearing itself introduces complications in the form of uncontrolled environmental conditions and stresses. Finally, there is good evidence to suggest that neonate caterpillars can exploit host-plant buds as food as soon as the bud scales begin to open (Stokoe 1948). In this experiment, I collected foliage in the field later, where leaves were small but recognisable in shape (Fig. S4). If the fitness effects of asynchrony on oak are particularly sudden and severe, foliage provided to the caterpillars, although young, may have been old enough to produce higher than expected levels of mortality. Nonetheless, these data provide informative comparisons across host-plant species at this early phenological stage.

Lab rearing assays capture one aspect of dietary ecology: the palatability of the host-plant. In nature, different tree species and individuals at different stages of growth provide structurally different habitats, which might affect predation risk from vertebrates, parasitism, and susceptibility to adverse abiotic conditions. Although female winter moths seem unable to choose the species of the host tree they ascend, they do tend to select larger trees (Connell 2013; Graf et al. 1995; Kulfan et al. 2019). This could have taxonomic implications in many woodlands, where most mature trees are of a particular species, but may also imply that larger trees offer other inherent advantages for developing caterpillars—for example, reduced competition. It would certainly be possible, therefore, for extrinsic, non-dietary factors to differ sufficiently between host-plant species in the field, *such that performance in the lab ran counter to abundance observed in nature*. In other words, oak may still be the best host in the field, but not the best under lab conditions. Indeed, Wint (1983) showed that neonate winter moth caterpillars showed a strong innate preference for oak and apple foliage in choice experiments. However, field studies comparing the abundance of winter moth caterpillars or defoliation across different host-plant species have reported mixed results, with abundance generally being highest to mid-level on oak (see Table S2 and also: Shutt et al. 2019; Macphie et al. 2020). O’Donnell et al. (2019) found that abundance in the field and performance in captive rearing experiments (*N* = 60 / host-plant) were higher on oak than on other host-plants, but the extent to which abundance was greater on oak in the field *exceeded* the differences in lab performance. We are presented, then, with a complex array of interacting factors: for example, the reduced development time that we see in oak (Fig. 3 and 4) might also reduce exposure to predation, potentially compensating for any loss in mass and/or fecundity. Clarifying the paradox of these contradictions between lab and field studies, as well as directly quantifying the fitness effects of asynchrony across different host-plant species, are obvious directions for future work. Although these results are unusual in the extent to which caterpillar performance is poor on oak, and should be treated with caution, they nonetheless serve to illustrate that, across populations, there are a large number of common, alternative host-plant species on which performance is comparable or exceeds that on oak.

### Evidence of local adaptation to floral composition

A winter moth caterpillar hatching in spring faces two principal uncertainties: the species and the phenological stage of its host tree. Variation in either of these factors can significantly impact overall performance (Asch et al. 2007; Asch and Visser 2007; Feeny 1970, 1976; Tikkanen and Julkunen-Tiitto 2003; Wint 1983). Given the limited dispersal ability of females and that they seem unable to exert much, if any, host choice, we might expect populations to adapt to locally abundant host-plant species. Although I find quite clear evidence of divergence between the British winter moth populations studied in this experiment, these differences do not obviously conform to predictions we might make based on the character of the flora at each collection site (cf. Table 1 and Fig. 4).

The scant data already available does seem to suggest that under at least some circumstances, winter moth caterpillars perform better on locally prevalent host-plants. For example: on birch *Betula* spp. in Scandinavia (Belsing 2015; Lavola et al. 1998); on bird-cherry *Prunus padus* in Karelia (Tikkanen et al. 2000; Tikkanen and Lyytikäinen-Saarenmaa 2002); and on heather *Calluna* from heathland populations (Kerslake and Hartley 1997)*.* It has even been suggested that synchronisation to host-plant phenology is so important for fitness that the moths can adapt to a particular host-plant individual (Dongen et al. 1997) and that this need has driven the evolution of flightlessness in the females—to inhibit gene flow and encourage extremely local adaptation (Feeny 1970). However, genetic studies have shown that the dispersal of the winged male winter moths results in significant gene flow among winter moth populations which would act to inhibit spatial differentiation (Dongen et al. 1998; Legget et al. 2011). Taken together with my data, this perhaps suggests that local adaptation to host-plant availability in this species can occur, but at a larger spatial scale than I considered here or in other situations of extreme population isolation (e.g., islands, see Kerslake and Hartley 1997). Determining the scale at which winter moth populations may be adapted to local flora would necessitate further investigation with higher levels of population replication.

In nature, relative phenological synchronisation between caterpillars and their host-plants may be a more significant determinant of overall fitness than the inherent palatability of a host-plant (Asch et al. 2007; Tikkanen and Julkunen-Tiitto 2003)—the most abundant host may not necessarily be the best host. If the performance effects of asynchrony vary among host-plants, then selection favouring feeding on the host-plant on which the effects of asynchrony are least severe (or the rewards of synchrony are greatest) may outweigh pressure to optimise performance on the most abundant host-plant species.

### Polyphagy as buffering in an uncertain environment

By hatching in early spring and exploiting the young foliage of their host-plants, winter moth caterpillars occupy a narrow *phenological niche.* This is driven by selective pressures arising from variation in host palatability with time—the cost of asynchrony (Asch et al. 2007; Tikkanen and Julkunen-Tiitto 2003). Trophic generalism may be one mechanism by which they persist in their complex, heterogeneous ecological environment—consisting of many host-plant species, each varying in leafing phenology, unevenly spatially distributed and unequally palatable. By decreasing specificity in one aspect of their niche, winter moth caterpillars are able to specialise on a narrow phenological niche. Although trophic generalism is maintained throughout the distribution of the winter moth, we see indications that performance on particular host-plant species can be modulated in certain environments and populations, perhaps increasing fitness on locally abundant hosts while still being able to persist on many. Although trophic generalism prohibits specialisation on one host-plant, it likely results in a higher geometric mean fitness over time (Childs et al. 2010; Dempster 1955) because of the substantial fitness costs associated with asynchrony (Weir 2022).

The evidence I have presented here suggests that throughout its range, there are many plant species acceptable to winter moth caterpillars, on which performance is at least comparable to oak (even when treating the very poor results on oak with caution). This ability to effectively utilise a very large range of host-plant species might act as a diversified bet-hedging strategy and ameliorate the negative effects of asynchrony with bud burst on any one host-plant species (Weir 2022). To assess this potential mechanism for buffering mismatch, future studies should seek to directly test its operation in nature. An obvious testable prediction might be that in years of high mean asynchrony, winter moth populations should perform better in mixed versus low diversity woodlands, or should experience shallower inter-annual fluctuations in population size. The results of such experiments would be of far-reaching applicability and interest, beyond the narrow context of this one consumer species, because these principles are likely to generalise very widely.

The inherent resilience of the winter moth to a temporally uncertain niche and asynchrony with any one particular host-plant—as evinced by its success and abundance—might help buffer their populations against future climatic changes affecting phenology, and contribute to the stability of the wider ecosystem of which this species forms a crucial part. Beyond the winter moth, trophic generalism in consumers has the potential to buffer mismatch in a range of phenologically synchronised systems. In insectivorous birds—another heavily researched system—we still have a relatively incomplete understanding of how variation in dietary composition can affect fitness and how these effects vary across time (Macphie 2022). The example of the winter moth challenges us to revaluate the idea that phenological asynchrony is uniformly and severely negative for fitness. It serves to illustrate how critically important it is that we consider the wider ecological context of a species before we can expect to make robust projections as to the effects of climate change on their populations, or on those of species with which they interact.

## Supplementary Information

Below is the link to the electronic supplementary material.

Supplementary file1 (DOCX 816 KB)

Supplementary file2 (DOCX 444 KB)

Supplementary file3 (DOCX 18303 KB)

Supplementary file4 (XLSX 26 KB)

Supplementary file5 (XLSX 276 KB)

## Data Availability

Data used in this study are included as supplementary material and are also available through the Figshare repository (DOI: 10.6084/m9.figshare.27679935).

## Code availability

The data produced in this study were analysed with custom code using the *R* packages lme4 and MCMCglmm.

## Declarations

### Highlighted student research

This paper presents a novel experimental and conceptual perspective on an emblematic study system, suggesting why we fail to see widespread negative effects of phenological mismatch in nature.
