# Supplementary material for: Trophic generalism in the winter moth: a model species for phenological mismatch
Source: Oecologia. 2024 Nov 20;206(3-4):225–39. doi: 10.1007/s00442-024-05629-5 (PMC11599306; doi:10.1007/s00442-024-05629-5)
Supplement: Supplementary file 1 — Supplementary file1 (DOCX 816 KB) [file 442_2024_5629_MOESM1_ESM.docx]

**Appendix 1: Pupal mass, fecundity, and estimating fitness in the winter moth *Operophtera brumata* (Lep.: Geometridae)**

**Introduction**

Pupal mass in insects can be a reliable predictor of adult female fecundity (Heisswolf et al., 2009) and is therefore a very tractable measure of performance under different conditions—larger larvae produce larger pupae which yield larger females containing greater quantities of ova. Indeed, in the sizeable literature on the ecology and biology of the winter moth, variation in pupal mass across treatment groups is frequently interpreted as indicative of differences in fitness (see for example: Feeny, 1970; Wint, 1983; Kirsten and Topp, 1991; Tikkanen and Lyytikäinen-Saarenmaa, 2002; Belsing, 2015). In addition, if pupal mass falls below a certain level viability may be affected (Wint, 1983). Across insect species, both the slope of the mass-egg relationship (the correlation coefficient) and the intercept can vary—and can be affected within a species by environmental conditions.

In analysing this relationship, it is possible that we might find these parameters differing, plastically or adaptively, within a species, under different conditions, to optimise fitness (for example, by producing more ova per unit mass on a given host-plant). Methodologically there is further a distinction to be drawn between potential fecundity (the number of mature oocytes or eggs in the reproductive tract) and realised fecundity (the number of fertile ova laid by a female during her lifetime)—the relationship between pupal mass and the latter is often far less precise (see for example Heisswolf *et al*., 2009). In the winter moth, only three studies have attempted to quantify the mass-fecundity relationship experimentally. Both Holliday (1977) and Rubtsov and Utkina (2011) trapped wild female moths in winter on tree trunks and measured their fresh mass and potential fecundity. The data obtained by the former are also reported by Singer and Parmesan (2010), though mistakenly attributed to a later paper (Holliday, 1985). Roland and Myers (1987) also trapped and weighed wild caught females, then back-calculated pupal mass, and estimated potential fecundity by dissection.

Here, I analysed the relationship between fecundity and pupal mass in the winter moth using my own data and that reported by the other studies described above. I tested if the relationship varied by population and if it was affected by the host-plant on which the caterpillar was reared. From this relationship I show how a metric of fitness can be estimated when caterpillar survival to pupation and pupal mass are both measured.

**Pupal mass as a predictor of fecundity, and the effects of geographical and trophic factors**

**Methods**

I measured the fresh mass of winter moth pupae raised in this captive rearing experiment one month after pupation (+/- 5 days). Pupae were then stored at 21°C until 1 September 2020, when they were placed outdoors in ambient temperature (Stirlingshire, UK; 56.069°N, -3.767°E) in sealed containers under an open canopy which provided shade. After female eclosion later in winter (December), they were killed in 75% ethanol and dissected. The potential fecundity of each individual was measured (no. oocytes/female). To analyse the mass-fecundity relationship in these data I fitted a general linear mixed effects model in the *R* v. 4.0.3 package MCMCglmm (Hadfield, 2010). I tested for differences in the mass-fecundity relationship among host-plants by allowing for a random slope and intercept across host-plants (Model 1. Response: Fecundity; Explanatory Fixed effects: Pupal Mass, Population; Random effect: Pupal Mass:Host-plant, with random slopes. Default priors, 500000 iterations with 250000 burn-in, thinning every 50 iterations). Pupal mass was mean centred at 25mg.

In addition to my own data, I used the *R* package metaDigitise (Pick et al., 2018) to extract the mass-fecundity data from the relevant figures in three previous papers which measured the fecundity and mass of winter moth individuals (Holliday, 1977; Roland and Myers, 1987; Rubtsov and Utkina, 2011). I combined these data with my own to generate an overall model and test for: (i) any differences in the mass-fecundity relationship between winter moth populations at different geographical sites; and, (ii) any significant difference in the mass-fecundity relationship between studies using adult female mass or pupal mass as proxies for fecundity (Model 2. Response: Fecundity; Explanatory Fixed effects: Pupal Mass, Dataset; Random effects: Host-plant. Default priors, 500000 iterations with 250000 burn-in, thinning every 50 iterations).

**Results and discussion**

Those studies which measure female mass, rather than pupal mass, unsurprisingly find that predicted fecundity at a mass of 25mg (the approx. mean pupal mass) is significantly higher (Holliday = 174.41 ova/female, CIs: 163.92, 184.67; Rubtsov and Utkina = 159.43, CIs: 154.18, 164.26 vs Roland and Myers = 140.23, CIs: 133.53, 164.26; Weir = 143.68, CIs: 139.24, 148.12) (Figure A1.1). This may be accounted for by individuals undergoing a loss of mass either during the period of development from summer to eclosion in winter (e.g. through water loss) or during the process of eclosion itself, which involves shedding the pupal case. As pupal/female mass increases in my data, fecundity increases significantly (slope = 9.81, CIs: 9.26, 10.34). The mass-fecundity slope does not differ significantly between my study and the other datasets (Holliday = 10.20, CIs: 9.46, 10.99; Roland and Myers = 9.57, CIs: 8.88, 10.25; Rubtsov and Utkina = 9.46, CIs: 9.04, 9.89).

Taking my own data separately, point estimates of the among host-plant species variance in the intercept (26.24, CIs: 0.00, 108.51; intercept mean-centred at 25mg) and slope (0.86, CIs: 0.00, 3.16) of the mass-fecundity relationship are small and the lower bounds of the credible intervals approach zero, suggesting these are non- or marginally significant. Pupal or adult female mass is therefore a consistent predictor of potential fecundity, with no significant geographical or host-specific variation across these data (Figure A1.1).

**Estimating fitness in the winter moth**

Factors such as survival, pupal mass, or development time are informative with regard to caterpillar performance on different host-plants, but are only a few of the many facets affecting overall fitness. Interpreting how these interact can be difficult: how does performance on a host species which yields high mortality but high pupal mass compare with one resulting in low mortality but low final pupal mass? Ideally, we could measure overall fitness in the field in each case, or as close an approximation to this as possible, to infer which is the optimal host-plant. Below, I develop a metric of absolute fitness in the winter.

Assuming the pupal mass-fecundity relationship is linear, we can predict the fecundity of a moth—a more reliable signpost of fitness—from its pupal mass using the standard equation of a straight line:

*y* = *mx* + *c*

As:

Fecundity = *m*_mf_ * Mass + *c*_mf_

Where *m*_mf_ is the posterior of the slope of pupal mass and potential fecundity found in my own data, Mass is the pupal mass in a given treatment group, and *c*_mf_ is the intercept of the mass-fecundity relationship. Using the posterior distributions of each of these terms from Bayesian models of my own data, I obtain a posterior for the predicted fecundity of an individual for a given pupal mass.

I then combine the predicted fecundity and the posterior of the probability of survival to give an estimate of fitness:


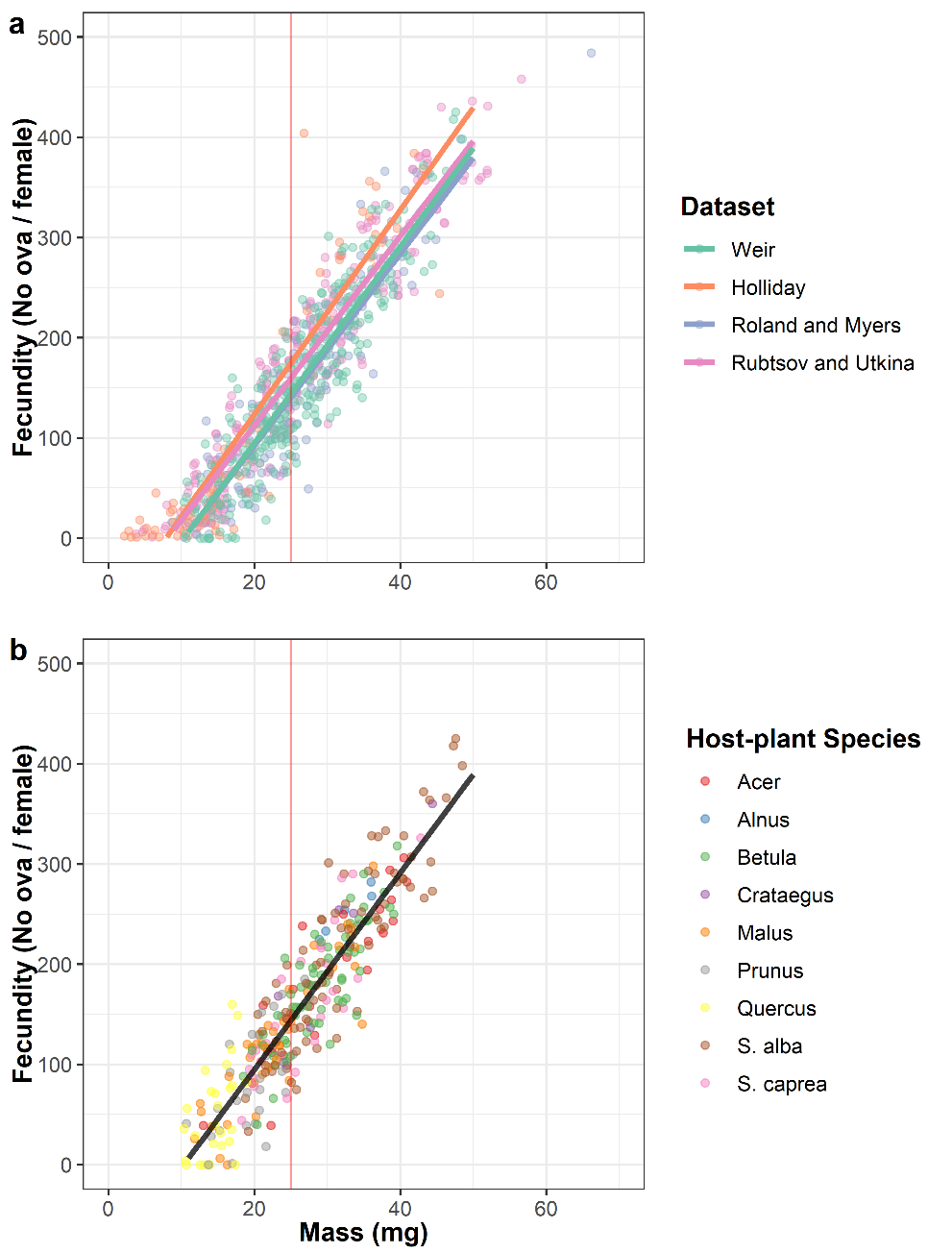


**Figure A1.1.** Relationship of winter moth pupal mass to potential female fecundity across (a) different studies and (b) across different host-plants in my own data. Regression line shows mean estimates for (a) each study (Slopes: Weir = 9.81, CIs: 9.26, 10.34; Holliday = 10.20, CIs: 9.46, 10.99; Roland and Myers = 9.57, CIs: 8.88, 10.25; Rubtsov and Utkina = 9.46, CIs: 9.04, 9.89) and (b) overall in the data from this study (slope = 9.81, CIs: 9.23, 10.32). Data in (a) were derived from Weir (this study), Holliday (1977), Roland and Myers (1987), and Rubtsov and Utkina (2011). Weir and Roland and Myers report fresh pupal mass, while Holliday and Rubtsov and Utinka use fresh female mass. Data in (b) were derived solely from the present study.

Fitness = Fecundity * Probability of Survival

Or, more completely:

Fitness = (*m*_mf_ * Mass + *c*_mf_) * (Survival)

The estimate of absolute fitness here is therefore given as the *predicted ova per female in a given treatment group*. From the posterior distributions it is possible to calculate a mean value and 95% HPD interval on this estimate of fitness.

**References**

Belsing, U. (2015). *The survival of moth larvae feeding on different plant species in northern Fennoscandia*. Lund University.

Feeny, P. (1970). Seasonal changes in oak leaf tannins and nutrients as a cause of spring feeding by winter moth caterpillars. *Ecology* **51**:565–581.

Hadfield, J. D. 2010. MCMC methods for multi-response generalized linear mixed models: the MCMCglmm R package. *Journal of Statistical Software* **33**:1–22.

Heisswolf, A., T. Klemola, T. Andersson, and K. Ruohomäki. (2009). Shifting body weight-fecundity relationship in a capital breeder: maternal effects on egg numbers of the autumnal moth under field conditions. *Bulletin of Entomological Research* **99**:73–81.

Holliday, N. J. (1977). Population ecology of winter moth (*Operophtera brumata*) on apple in relation to larval dispersal and time of bud burst. *The Journal of Applied Ecology* **14**:803.

Holliday, N. J. (1985). Maintenance of the phenology of the winter moth (Lepidoptera: Geometridae). *Biological Journal of the Linnean Society* **25**:221–234.

Kirsten, K., and W. Topp. (1991). Acceptance of willow‐species for the development of the winter moth, *Operophtera brumata* (Lep., Geometridae). *Journal of Applied Entomology* **111**:457–468.

Pick, J. L., S. Nakagawa, and D. W. A. Noble. (2018). Reproducible, flexible and high-throughput data extraction from primary literature: the metaDigitise R package. https://doi.org/10.1101/247775.

Roland, J., and J. H. Myers. (1987). Improved insect performance from host-plant defoliation: winter moth on oak and apple. *Ecological Entomology* **12**:409–414.

Rubtsov, V., and I. A. Utkina. (2011). Long-term dynamics of *Operophtera brumata* L. in the oak stands of forest-steppe. *Contemporary Problems of Ecology* **4**:36–45.

Singer, M. C., and C. Parmesan. (2010). Phenological asynchrony between herbivorous insects and their hosts: signal of climate change or pre-existing adaptive strategy? *Philosophical Transactions of the Royal Society B: Biological Sciences* **365**:3161–3176.

Tikkanen, O.-P., and P. Lyytikäinen-Saarenmaa. (2002). Adaptation of a generalist moth, *Operophtera brumata*, to variable budburst phenology of host plants. *Entomologia Experimentalis et Applicata* **103**:123–133.

Wint, W. (1983). The role of alternative host-plant species in the life of a polyphagous moth, *Operophtera brumata* (Lepidoptera: Geometridae). *Journal of Animal Ecology* **52**:439–450.
