# Supplementary material for: Trophic generalism in the winter moth: a model species for phenological mismatch
Source: Oecologia. 2024 Nov 20;206(3-4):225–39. doi: 10.1007/s00442-024-05629-5 (PMC11599306; doi:10.1007/s00442-024-05629-5)
Supplement: Supplementary file 2 — Supplementary file2 (DOCX 444 KB) [file 442_2024_5629_MOESM2_ESM.docx]

**Appendix 2: The effects of mixed brood cultures on the analysis of caterpillar performance and geographical divergence**

A weakness of the experimental design employed in this study was that, for the sake of logistical simplicity, several offspring from a single brood (all offspring of one female) were reared across each mixed brood culture of 20 individuals. This made it impossible to later identify which individual belonged to which brood. Caterpillars from the same brood will likely be more similar to one another in terms of their performance across host-plants than to other members of the population, due to genetic similarity and maternal effects.

In the context of the experimental design and analysis presented here, the variance for a given population may be under-estimated, relative to the real value in nature, because some of the individuals within each culture are related. And, since individual identity is not tracked, this is cannot be controlled for in the statistical analysis. In addition, where similar brood compositions are used across different experimental treatments it may be expected to bias effects towards the null (i.e. no effect). Alternatively, biological processes such as diversified bet-hedging among the offspring of individual females may mean that among brood variances are small relative to among offspring differences—in this case, we would not expect the results to be biased in the way outlined above.

For analyses conducted at the culture level (e.g. survival) it is possible to include the contributing broods as a multi-membership random term and to estimate the brood effect, but for analyses that must be conducted at the individual level (e.g. mass at pupation) this cannot be done. Therefore, to assess the sensitivity of the statistical inferences presented in this study to brood effects of different magnitudes I conducted extensive simulations.

To recap, the two main aims of this study were:

1. To quantify variation in caterpillar performance among nine host-plant species
2. To quantify host-by-population variation in caterpillar performance (suggesting geographical divergence)

Based on the anticipated impact of among-brood variance on model parameters, it follows that if the variation in performance among broods is substantial then this design and analysis will tend to have a high false positive rate for detecting among population differences, and reduced power to detect among host-plant and host-by-population effects. The aim of the simulations presented here was to assess the sensitivity of these effects, under the focal experimental design, with different magnitudes of among-individual and among-brood variance.

**Simulated data and analyses**

To explore the implications of the experimental design presented here on the expected outcome of the analyses (variance estimates and false positive rates), I generated a simulation of the study in *R* (assuming a Gaussian response) and manipulated the variance of each of the main parameters. The data and model structure followed that used in the study, including the difference in brood number between Edinburgh and the remaining three populations. Across simulations I assessed the sensitivity of inferences to different magnitudes of variance among individuals, broods, populations, host-plants, and host-by-population interactions (Table A2.1). The among culture variance was set at 0.005 for all simulations. The simulation code is provided at the end of this appendix. For each parameter combination I conducted 500 simulations. For each of the variance terms that are critical to the hypotheses being tested (population, host-plant, host-by-population) I quantified: the median variance estimate across simulations to assess bias; and, the proportion of simulations returning a significant p-value (< 0.05, based on a likelihood ratio test) to assess the false positive (type I error) rate.

From these simulations we can see that under certain circumstances the design can produce a high rate of false positive effects of population (Fig. A2.1). However, this only occurs in a limited set of situations, with the severity of the issue increasing as the among-brood variation increases relative to among-individual variation. Variance estimates have broad confidence intervals, but are generally not significantly different from the true value in each simulation. For the remaining parameters—including, crucially, the host-plant by population interaction term—the design and model are very conservative. This is even the case when among-brood variation is much greater than among-individual variation (a situation which I think unlikely to be the case in nature, and which is inherently more likely, under this design, to produce spurious population effects).

**Conclusions**

Although these simulations suggest that, under some circumstances, the experimental design is prone to false positives for an effect of population, none of the results presented in this paper show such an effect. More importantly, the simulations demonstrate that the design and model generate very conservative estimates of the variance and significance of both the effect of host-plant species (**Aim A**) and the host-plant by population interaction (**Aim B**). This evidence, taken collectively, demonstrates that this experimental design is appropriate for addressing the stated aims of this study.

**Table A2.1.** Parameters used in simulations of the study design. Each combination (‘Scenario’) of among population, among host-plant, and host-plant by population variance used in the simulations are assigned a lettered code, referred to in Figure A2.1.

| **Variance** | | | | | |  |
| --- | --- | --- | --- | --- | --- | --- |
| Among individual | Among females/  broods | Among rearing cultures | Among populations | Among host-plants | Host-plant:  population  inter. | Scenario |
| 1 | 1 | 0.05 | 0 | 0 | 0 | *a* |
| 1 | 1 | 0.05 | 10 | 0 | 0 | *b* |
| 1 | 1 | 0.05 | 0 | 10 | 0 | *c* |
| 1 | 1 | 0.05 | 0 | 0 | 10 | *d* |
| 1 | 1 | 0.05 | 10 | 10 | 0 | *e* |
| 1 | 1 | 0.05 | 0 | 10 | 10 | *f* |
| 1 | 1 | 0.05 | 10 | 0 | 10 | *g* |
| 1 | 1 | 0.05 | 10 | 10 | 10 | *h* |
| 10 | 1 | 0.05 | 0 | 0 | 0 | *a* |
| 10 | 1 | 0.05 | 10 | 0 | 0 | *b* |
| 10 | 1 | 0.05 | 0 | 10 | 0 | *c* |
| 10 | 1 | 0.05 | 0 | 0 | 10 | *d* |
| 10 | 1 | 0.05 | 10 | 10 | 0 | *e* |
| 10 | 1 | 0.05 | 0 | 10 | 10 | *f* |
| 10 | 1 | 0.05 | 10 | 0 | 10 | *g* |
| 10 | 1 | 0.05 | 10 | 10 | 10 | *h* |
| 1 | 10 | 0.05 | 0 | 0 | 0 | *a* |
| 1 | 10 | 0.05 | 10 | 0 | 0 | *b* |
| 1 | 10 | 0.05 | 0 | 10 | 0 | *c* |
| 1 | 10 | 0.05 | 0 | 0 | 10 | *d* |
| 1 | 10 | 0.05 | 10 | 10 | 0 | *e* |
| 1 | 10 | 0.05 | 0 | 10 | 10 | *f* |
| 1 | 10 | 0.05 | 10 | 0 | 10 | *g* |
| 1 | 10 | 0.05 | 10 | 10 | 10 | *h* |
| 10 | 10 | 0.05 | 0 | 0 | 0 | *a* |
| 10 | 10 | 0.05 | 10 | 0 | 0 | *b* |
| 10 | 10 | 0.05 | 0 | 10 | 0 | *c* |
| 10 | 10 | 0.05 | 0 | 0 | 10 | *d* |
| 10 | 10 | 0.05 | 10 | 10 | 0 | *e* |
| 10 | 10 | 0.05 | 0 | 10 | 10 | *f* |
| 10 | 10 | 0.05 | 10 | 0 | 10 | *g* |
| 10 | 10 | 0.05 | 10 | 10 | 10 | *h* |


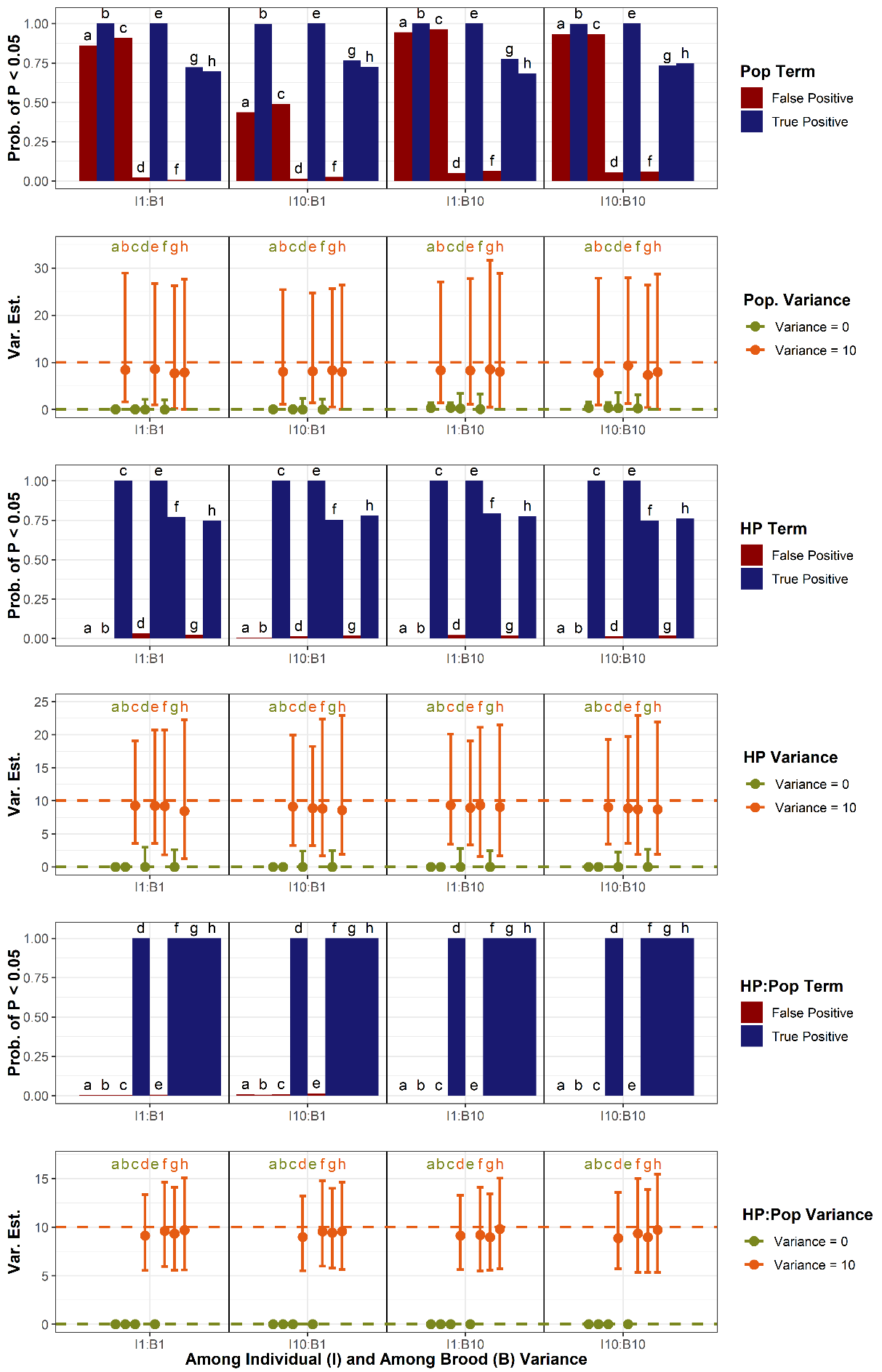


**Figure A2.1.** True and false positive rate of term significance and the variance estimates (±95%) in simulated analyses, shown for Population term (Pop), Host-plant term (HP), and Host-plant by population interaction term (HP:Pop). For details of each simulated scenario (lettered *a* to *h*), see Table A2.1. Each scenario is shown simulated with four levels of among-individual and among-brood variance.

**Annotated R Code for Simulations**

##Step 1 – Set-up the structure of the experiment and dataset

population<-as.factor(rep(rep(1:4,each=100),9))

#population1==Edi

tray<-as.factor(rep(1:180,each=20))

hostplant<-as.factor(rep(1:9,each=400))

female<-rep(NA,3600)

female[1:100]<-female[401:500]<-female[801:900]<-female[1201:1300]<-female[1601:1700]<-female[2001:2100]<-female[2401:2500]<-female[2801:2900]<-female[3201:3300]<-1:100

#Set-up female sampling design for the other trays (i.e. ‘cultures’) and sites. The same female structure across all trays and host-plants

females2<-c(126:141,126:129)

females3<-c(142:155,142:147)

females4<-c(156:174,156)

plusx<-100

female[plusx+1:100]<-female[plusx+401:500]<-female[plusx+801:900]<-female[plusx+1201:1300]<-female[plusx+1601:1700]<-female[plusx+2001:2100]<-female[plusx+2401:2500]<-female[plusx+2801:2900]<-female[plusx+3201:3300]<-rep(females2,5)

plusx<-200

female[plusx+1:100]<-female[plusx+401:500]<-female[plusx+801:900]<-female[plusx+1201:1300]<-female[plusx+1601:1700]<-female[plusx+2001:2100]<-female[plusx+2401:2500]<-female[plusx+2801:2900]<-female[plusx+3201:3300]<-rep(females3,5)

plusx<-300

female[plusx+1:100]<-female[plusx+401:500]<-female[plusx+801:900]<-female[plusx+1201:1300]<-female[plusx+1601:1700]<-female[plusx+2001:2100]<-female[plusx+2401:2500]<-female[plusx+2801:2900]<-female[plusx+3201:3300]<-rep(females4,5)

female<-as.factor(female)

hostbypop<-as.factor(as.numeric(as.factor(paste(hostplant, population))))

##Step 2 – Set-up parameters.

#generate model data-frame to fill in with results of sims

sims<-data.frame(ind=numeric(), brood=numeric(), culture=numeric(), pop=numeric(), hp=numeric(), hp.pop=numeric(), fp_pop=numeric(), fp_hp=numeric(), fp_hp.pop=numeric(), var_m_pop=numeric(), var_m_hp=numeric(), var_m_hp.pop=numeric(), var_l_pop=numeric(), var_u_pop=numeric(), var_l_hp=numeric(), var_u_hp=numeric(), var_l_hp.pop=numeric(), var_u_hp.pop=numeric())

#load data-frame of test variances to plug into models, see attached file **sim_data**#

#set-up row no. from data-frame of test variances

r<-1

#specify parameters used for each component of simulation, drawing values from each row in sim_data

individualvar<-sim_data[r,1]

femalevar<-sim_data[r,2]

trayvar<-sim_data[r,3]

populationvar<-sim_data[r,4]

hostplantvar<-sim_data[r,5]

hostbypopulationvar<-sim_data[r,6]

##Step 3 - Simulate data

resp<-0+rnorm(4,0,sqrt(populationvar))[population]+rnorm(9,0,sqrt(hostplantvar))[hostplant]+rnorm(36,0,sqrt(hostbypopulationvar))[hostbypop]+

rnorm(174,0,sqrt(femalevar))[female]+rnorm(180,0,sqrt(trayvar))[tray]+rnorm(3600,0,sqrt(individualvar))

##Step 4 - Run model

library(lme4)

#model<-lmer(resp~1+(1|population)+(1|hostplant)+(1| hostbypop)+(1|tray))

#summary(model)

#run 500 simulations

#create places to store accumulated data from repeated runs of model

pval_population<-c()

pval_hostplant<-c()

pval_hostbypop<-c()

varpop<-c()

varhost<-c()

varhostbypop<-c()

for (sim in 1:500){

resp<-0+rnorm(4,0,sqrt(populationvar))[population]+rnorm(9,0,sqrt(hostplantvar))[hostplant]+rnorm(36,0,sqrt(hostbypopulationvar))[hostbypop]+

rnorm(174,0,sqrt(femalevar))[female]+rnorm(180,0,sqrt(trayvar))[tray]+rnorm(3600,0,sqrt(individualvar))

model<-lmer(resp~1+(1|population)+(1|hostplant)+(1| hostbypop)+(1|tray))

model_nointer<-lmer(resp~1+(1|population)+(1|hostplant)+(1|tray))

model_nohost<-lmer(resp~1+(1|population)+(1| hostbypop)+(1|tray))

model_nopop<-lmer(resp~1+(1|hostplant)+(1| hostbypop)+(1|tray))

pval_hostbypop[sim]<-anova(model, model_nointer)["Pr(>Chisq)"][[1]][2]

pval_hostplant[sim]<-anova(model, model_nohost)["Pr(>Chisq)"][[1]][2]

pval_population[sim]<-anova(model, model_nopop)["Pr(>Chisq)"][[1]][2]

varhostbypop[sim]<-VarCorr(model)$hostbypop[1]

varhost[sim]<-VarCorr(model)$hostplant[1]

varpop[sim]<-VarCorr(model)$population[1]

}

par(mfrow=c(3,2))

##Plotting each effect, evaluating median estimates relative to true value, and false/true positive rates

hist(pval_population)

hist(varpop)

abline(v= populationvar,col="red")

hist(pval_hostplant)

hist(varhost)

abline(v= hostplantvar,col="red")

hist(pval_hostbypop)

hist(varhostbypop)

abline(v= hostbypopulationvar,col="red")

##To calculate proportion f/positives

sims[nrow(sims) + 1,] = c(individualvar, femalevar, trayvar, populationvar, hostplantvar, hostbypopulationvar, sum(pval_population<0.05)/500, sum(pval_hostplant<0.05)/500, sum(pval_hostbypop<0.05)/500, median(varpop), median(varhost), median(varhostbypop), quantile(varpop, c(0.05, 0.95)), quantile(varhost, c(0.05, 0.95)), quantile(varhostbypop, c(0.05, 0.95)))
