# Supplementary material for: Trophic generalism in the winter moth: a model species for phenological mismatch
Source: Oecologia. 2024 Nov 20;206(3-4):225–39. doi: 10.1007/s00442-024-05629-5 (PMC11599306; doi:10.1007/s00442-024-05629-5)
Supplement: Supplementary file 3 — Supplementary file3 (DOCX 18303 KB) [file 442_2024_5629_MOESM3_ESM.docx]

Table S1. The role of oak in the life of the winter moth: a selection of the phenological literature. There is a general consensus among widely cited studies that English oak *Quercus robur* is the most important (and preferred) host-plant of the winter moth in the field. Number of citations shown underlined after reference, obtained from Google Scholar (accessed 3 February 2023), provide a rough estimate of the influence of each publication on the field.

| **Views on the role of oak *Quercus robur* as a host-plant** |
| --- |
| “For example, winter moth (*Operophtera brumata* L., Geometridae) larvae develop into heavier pupae when fed on young oak leaves than when reared on hazel (*Corylus avellana* L.) or blackthorn (*Prunus spinosa* L.), two species commonly used as host plants in the field (G. R. Gradwell, personal communication)”  **Feeny (1970)**, 2404 |
| “Larvae of the winter moth are able to feed on a wide range of trees and shrubs, but they are especially abundant on oaks (*Quercus robur*), which they sometimes defoliate.”  **Varley *et al.* (1974)**, 1101 |
| “Most of these losses are related to the degree of asynchrony between larval eclosion and the bud burst of the primary food-plant (*Quercus robur*).”  **Wint (1983)**, 143 |
| “The winter moth *Operophtera brumata* is one of the most common forest insects in Central Europe. The larvae feed on the oak, *Quercus robur*, as well as on several other species of broad-leaved trees.”  **Kirsten and Topp (1991)**, 24 |
| “The synchrony between the emergence of larval winter moth (*Operophtera brumata* L.) and budburst of pedunculate oak (*Quercus robur* L.), its primary host (Wint, 1983), is critical for the moth’s success.”  **Buse and Good (1996)**, 171 |
| “*Operophtera brumata* L. [is] one of the most important herbivores of oak.”  **Dongen *et al.* (1997)**, 139 |
| “*Operophtera brumata* L. (Lepidoptera: Geometridae) is one of the most abundant insect herbivores on *Q. robur*.”  **Tikkanen and Julkunen-Tiitto (2003)**, 174 |
| “*Operophtera brumata* is a polyphagous moth that is able to feed on a range of tree and shrub species (Holliday, 1985). In the U.K., oak *Quercus* spp. (Fagaceae) and other deciduous trees are regarded as the optimal hosts of this insect (Feeny, 1970; Wint, 1983; Holliday, 1985), but *O. brumata* has also been recorded on heather *Calluna vulgaris* (Ericaceae) where it reaches outbreak densities (Picozzi, 1981; Kerslake *et al*., 1996).”  **Vanbergen *et al.* (2003)**, 55 |
| “Timing of egg hatching in *O. brumata* is itself under selection for synchronization with bud burst in oak trees (*Quercus robur*). Caterpillars of this moth rely on oak leaves for food, and although the oak trees have been opening their buds earlier, advancement in the date of egg hatching has been more extreme.”  “Our fitness estimations are based on the assumption that the optimal moment of egg hatch is determined by the moment of *Q. robur* bud burst. *O. brumata* is not a specialist species: it can also feed on leaves from tree species other than oak.”  **van Asch *et al.* (2007)**, 252 |
| “Pedunculate oak, *Quercus robur*, is a favoured host of winter moth”  **Singer and Parmesan (2010)**, 324 |
| “However, caterpillars [of the winter moth] are available to the birds only during a brief period in spring, as the larvae exploit the newly emerged leaves of their host trees (predominantly oak *Quercus* spp.; Varley and Gradwell 1958)”  **Hinks *et al.* (2015)**, 68 |
| “Pedunculate oak is described as the primary host plant of winter moth throughout its native range.”  **O’Donnell *et al.* (2019)**, n/a |

[SEE EXCEL FILE]

Table S2. Taxonomic coverage of the literature on winter moth caterpillar performance across host-plants. Studies are grouped by those which consider the effects of varying degrees of asynchrony on performance across host-plant species and those which consider performance at one time point only. Particularly in studies of synchrony, there is a clear bias towards oak *Quercus*. The inclusion of a host-plant species in a particular study is indicated by ✓. Taxa arranged alphabetically within higher groupings. Performance Metrics used are: S (survival); P (pupal mass); GR (growth rate); DI (development index); DT (development time); FAb (abundance across host-plants measured in the field); LP (larval choice experiment); FU (food utilisation or assimilation rate); FP (frass production); F (fecundity); and MO (mass of ova).

Table S3. Establishment dates of caterpillar cultures on each host-plant species assayed in the experiment. The phenology of each host-plant species differed in the field. For each experiment “Time 0” was taken as the timing of the first small leaves breaking/unfurling, with a recognisable shape. The treatment groups for each host-plant species therefore began on different calendar dates, as indicated above. Variation in the calendar date timing of each treatment was minimised as far as possible, so as not to confound host-plant effects with any effect of hatch timing, but was limited based on the observed phenology of each species in the field. The latest treatments were begun several days after the earliest treatments.

| **Host-plant Species** | **Date of Establishment** |
| --- | --- |
| *Acer* | 1 May |
| *Alnus* | 29 Apr |
| *Betula* | 1 May |
| *Crataegus* | 25 Apr |
| *Malus* | 29 Apr |
| *Prunus* | 29 Apr |
| *S. alba* | 29 Apr |
| *S. caprea* | 2 May |
| *Quercus* | 4 May |


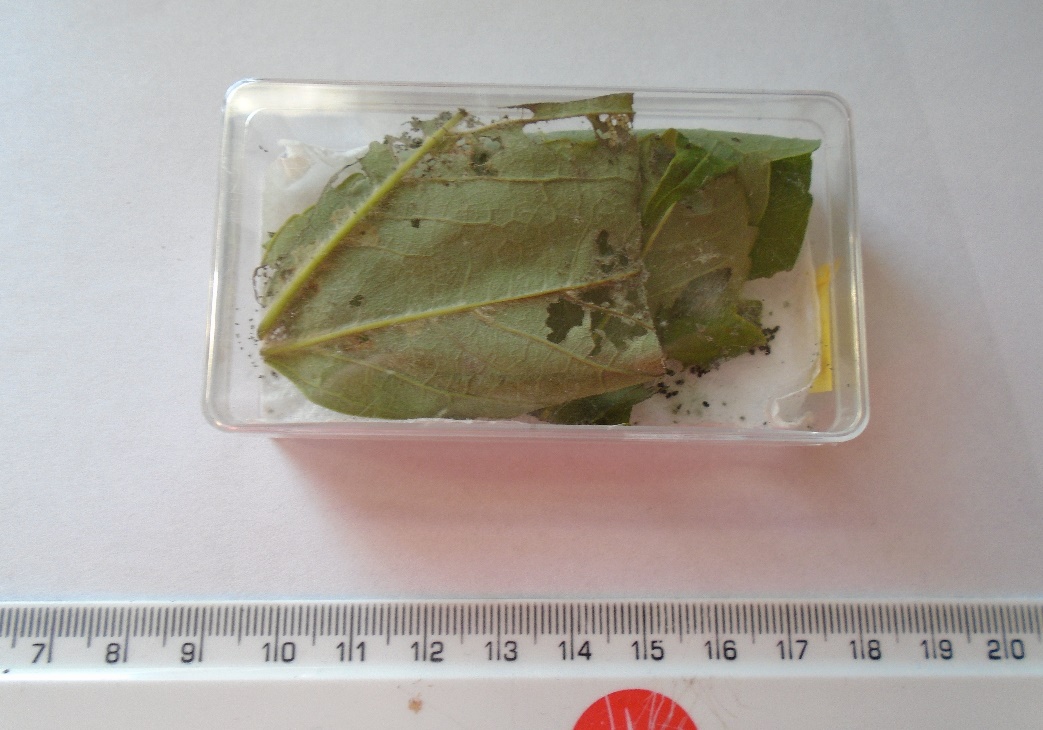

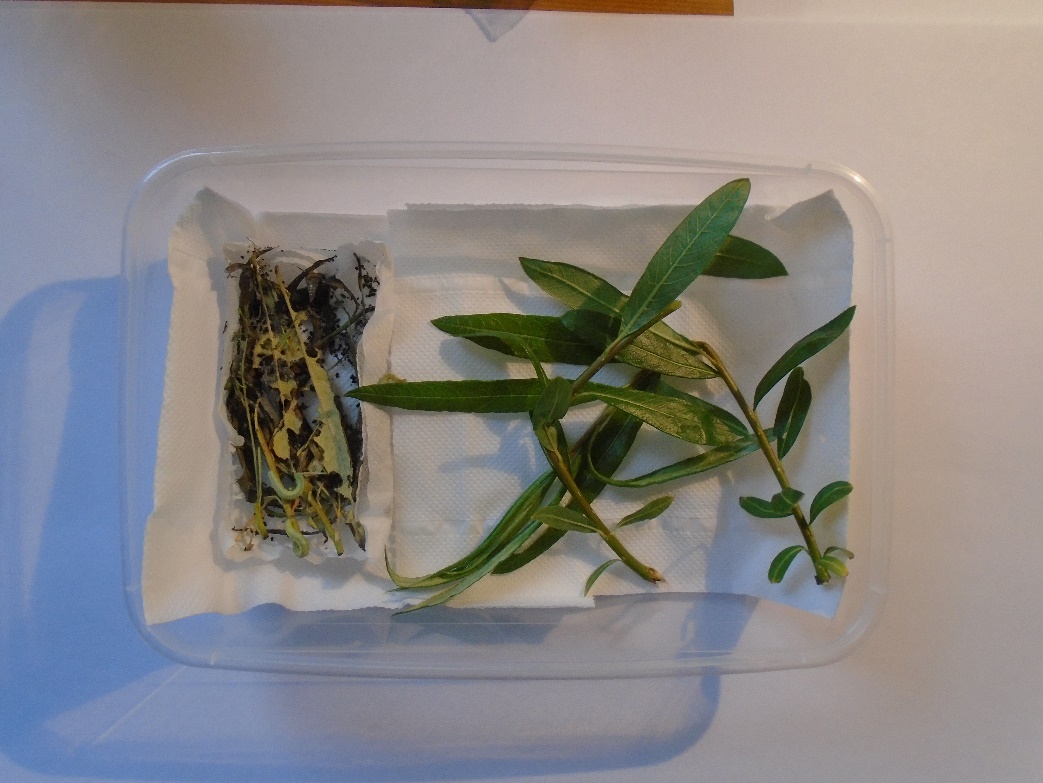


(a)

(b)

Figure S1. Caterpillar rearing containers. Caterpillars were housed when neonates in (a) small 75 x 50 x 15mm transparent plastic containers. When large enough, usually in the third instar, caterpillars were transferred to (b) larger 175 x 100 x 50mm disposable plastic food containers. Rearing containers were lined with tissue which was replaced each time new food was added. When new food was added at one end of the container the old food was left in to allow caterpillars to move onto the fresh plant material as and when they chose. Similarly, (b) when caterpillars were transferred to larger rearing containers, the whole contents of the smaller container were moved, and new food supplied for caterpillars to move off onto as they chose. Scale bar in (a) is in centimetres and millimetres.


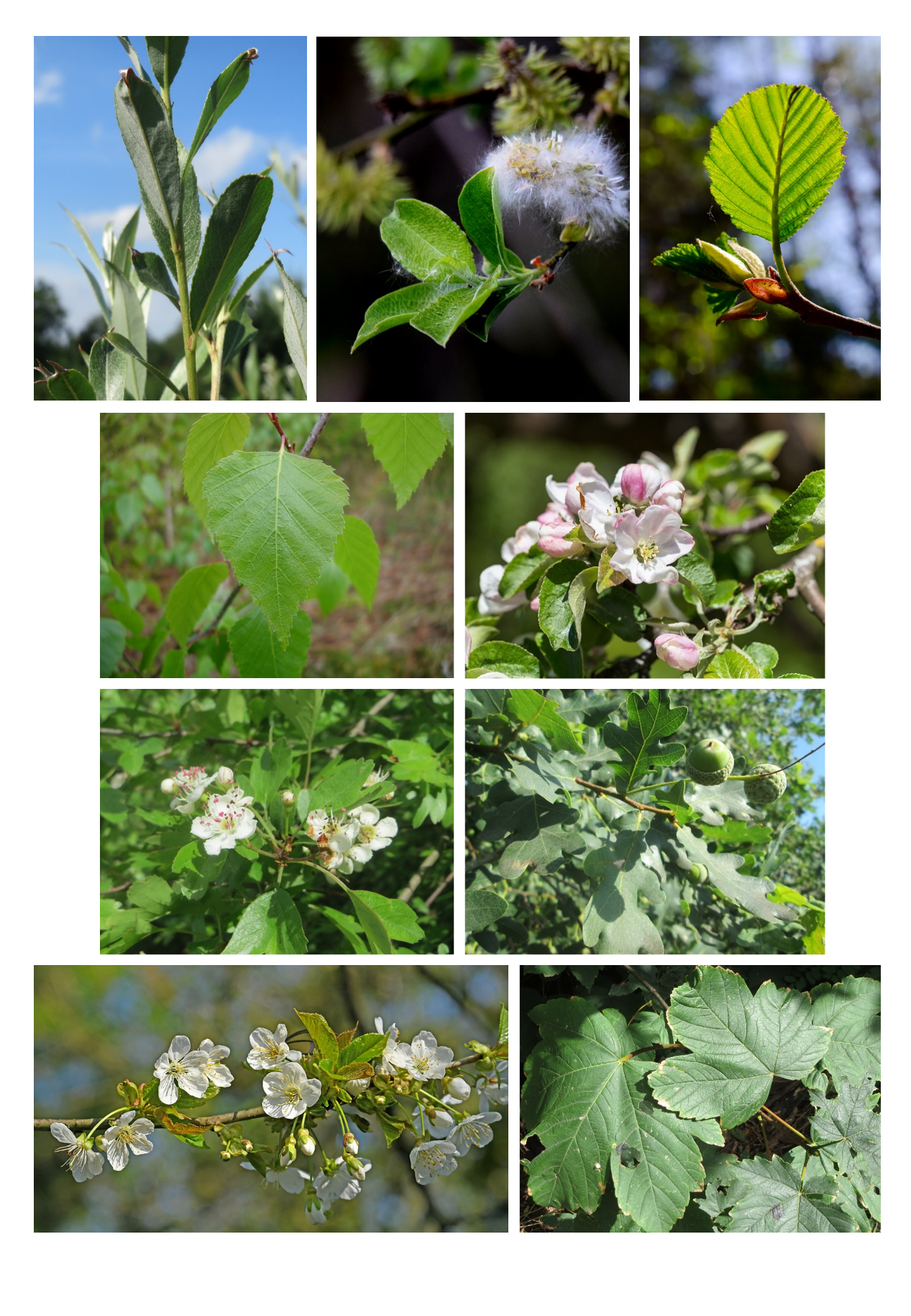


(a)

(b)

(c)

(d)

(e)

(f)

(g)

(h)

(i)

Figure S2. Host-plant species studied in the experiment. (a) Willow *Salix alba*, (b) Sallow *Salix caprea*, (c) Alder *Alnus glutinosa*, (d) Birch *Betula pendula*, (e) Apple Malus domestica, (f) Hawthorn *Crataegus monogyna*, (g) Oak *Quercus robur*, (h) Cherry *Prunus avium*, (i) Sycamore *Acer pseudoplatanus*. Image credits: Andreas Rockstein (a, f, g, I), Giuseppe Morlando (b), Tero Laakso (c), dragonfly201011 (d), Mariya Novikova (e), Karin Rogmann (h). Taken from Flickr.org, reproduced under a creative commons licence.


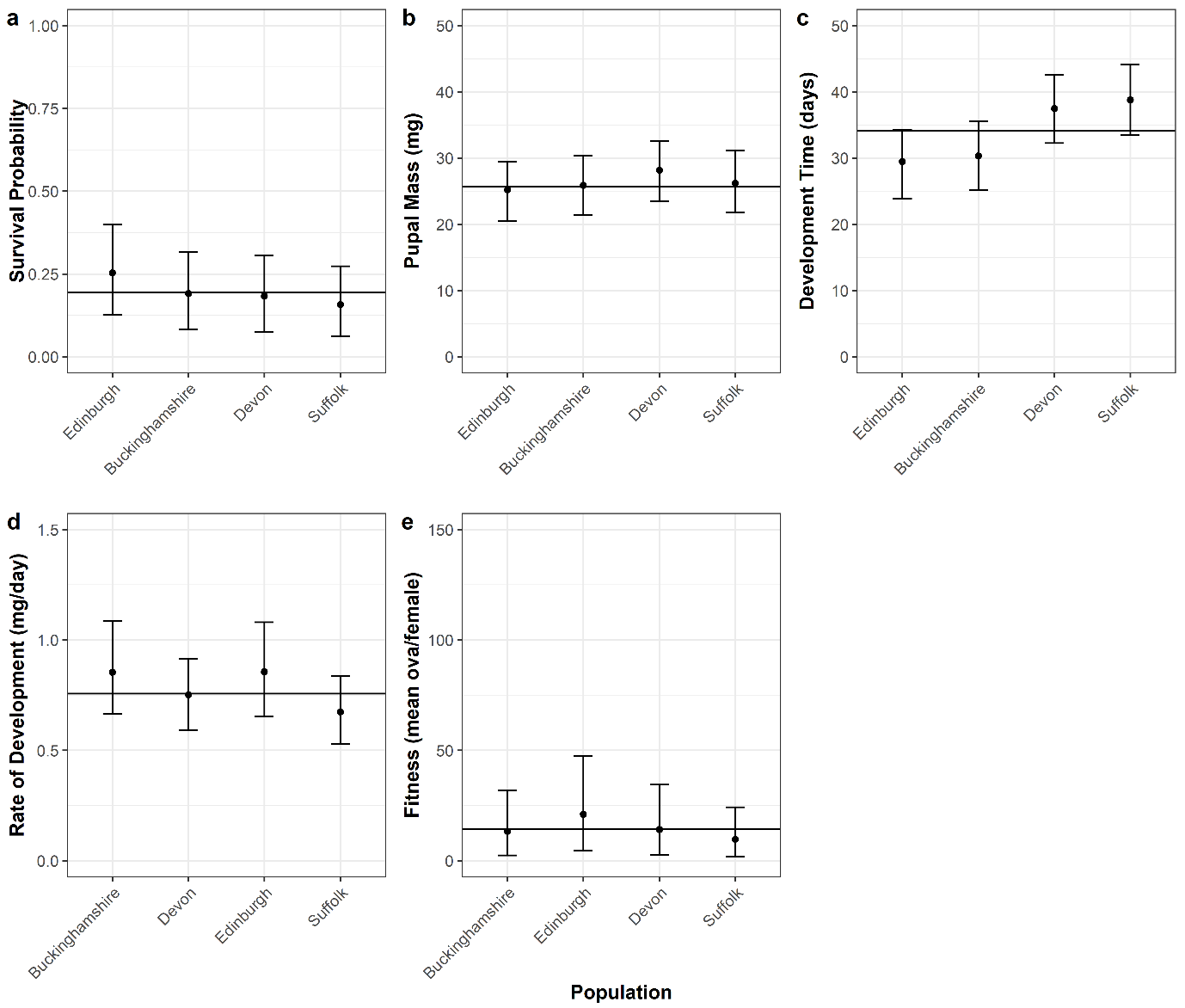


Figure S3. Overall winter moth performance in different populations across host-plants. Performance quantified as (a) survival probability, (b) pupal mass, (c) development time, (d) rate of development, and (e) estimated fitness. Mean estimates and 95% credible intervals shown. Global mean for each performance metric shown by solid line.


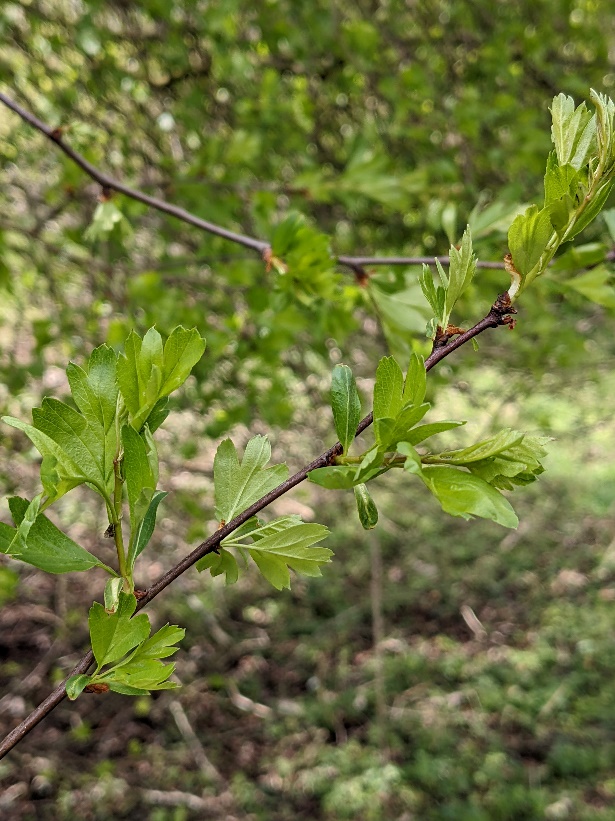

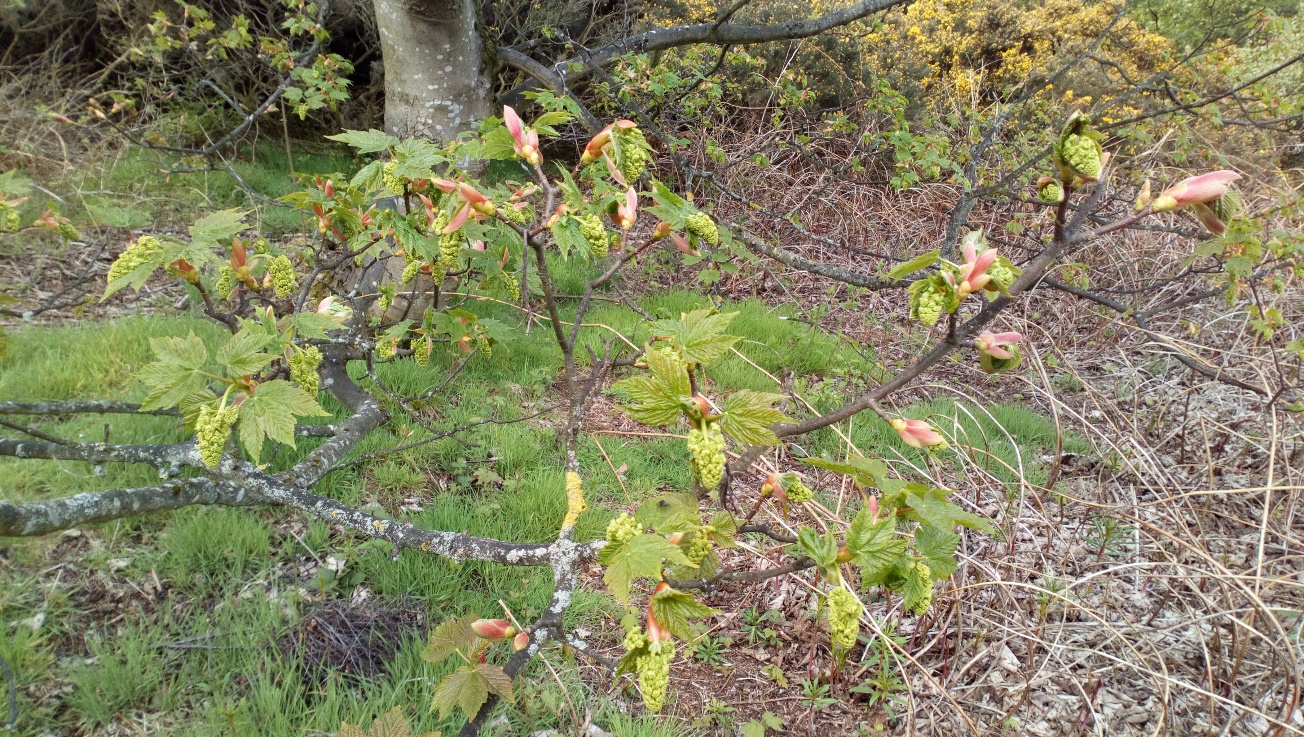

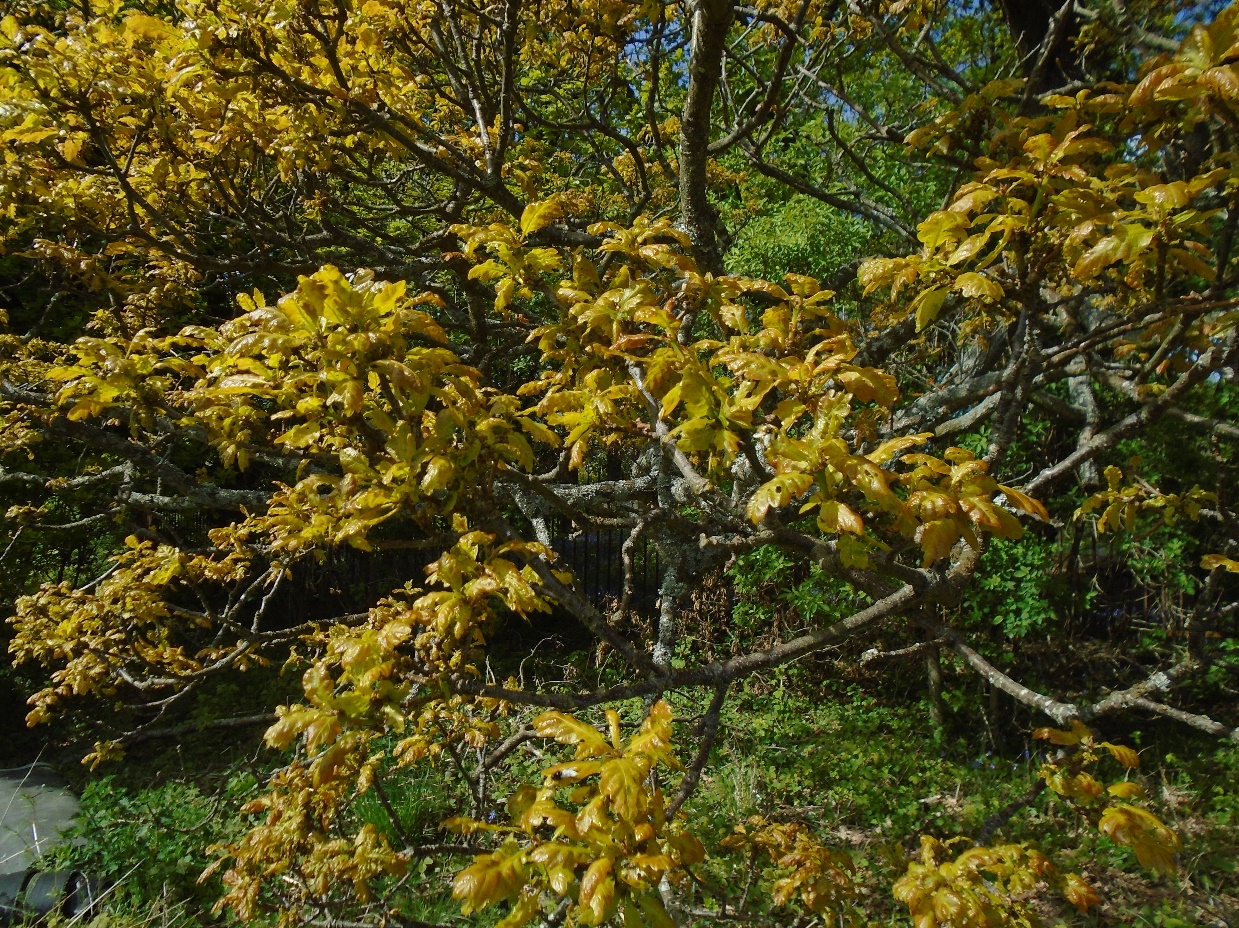


**(a)**

**(b)**

**(c)**

Figure S4. Examples of foliage at first collection. Species were collected at a common phenological stage, rather than based on calendar date, because this is more likely to be of functional significance for caterpillars and therefore standardised the effect of phenology on fitness across host-plant treatment groups. Leaf collection commenced when buds were broken and leaves were small but expanding, with a recognisable shape. Shown are examples of (a) hawthorn, (b) oak, and (c) sycamore.
